# Author Correction: The effects of mutational processes and selection on driver mutations across cancer types

**DOI:** 10.1038/s41467-020-15800-0

**Published:** 2020-04-20

**Authors:** Daniel Temko, Ian P. M. Tomlinson, Simone Severini, Benjamin Schuster-Böckler, Trevor A. Graham

**Affiliations:** 1 Evolution and Cancer Laboratory, Barts Cancer Institute, Barts and the London School of Medicine and Dentistry, Queen Mary University of London, Charterhouse Sq, London, EC1M 6BQ UK; 2 Department of Computer Science, University College London, London, WC1E 6BT UK; 3 Centre for Mathematics and Physics in the Life Sciences and Experimental Biology (CoMPLEX), University College London, Gower Street, London, WC1E 6BT UK; 4 Institute of Cancer and Genomic Sciences, University of Birmingham, Edgbaston, Birmingham B15 2TT UK; 5 Institute of Natural Sciences, Shanghai Jiao Tong University, Dong Chuan Road, Minhang District, Shanghai, 200240 UK; 6 Ludwig Institute for Cancer Research, University of Oxford, Old Road Campus Research Building, Roosevelt Dr, Oxford, OX3 7DQ USA

**Keywords:** Cancer genomics, Genome informatics, Cancer genomics

Correction to: *Nature Communications* 10.1038/s41467-018-04208-6, published online 10 May 2018.

The original version of this Article contained an error in author affiliations. Affiliation 1 incorrectly read Evolution and Cancer laboratory, Barts Cancer Institute, QMUL, Charterhouse Sq, London, EC1M 6BQ, UK.

This has been corrected in both the PDF and HTML versions of the Article.

