# Supplementary material for: The effects of mutational processes and selection on driver mutations across cancer types
Source: Nat Commun. 2018 May 10;9:1857. doi: 10.1038/s41467-018-04208-6 (PMC5945620; doi:10.1038/s41467-018-04208-6)
Supplement: Supplementary file 1 — Supplementary Information [file 41467_2018_4208_MOESM1_ESM.pdf]

The effects of mutational processes and selection on driver mutations across cancer types

Temko et al.

SUPPLEMENTARY INFORMATION

Supplementary Figures

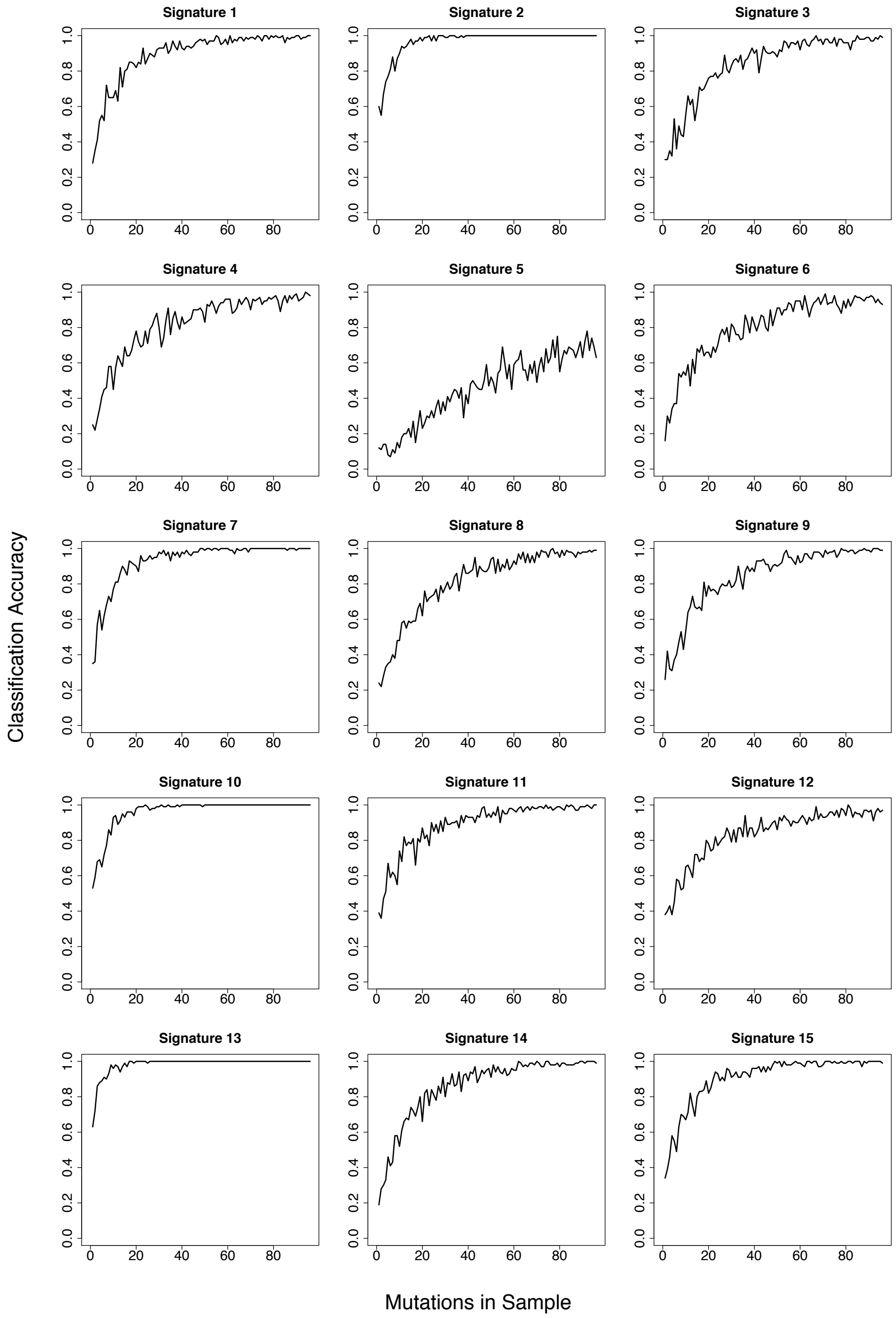

Supplementary Figure 1. Classification accuracies by number of informative mutations for each of signatures 1 to 15 as the true generating signature

Plots show the proportion of 100 simulations where signature assignment was successful (over half of the regression weight assigned to the true generating mutational signature) in simulated mutation data sets.

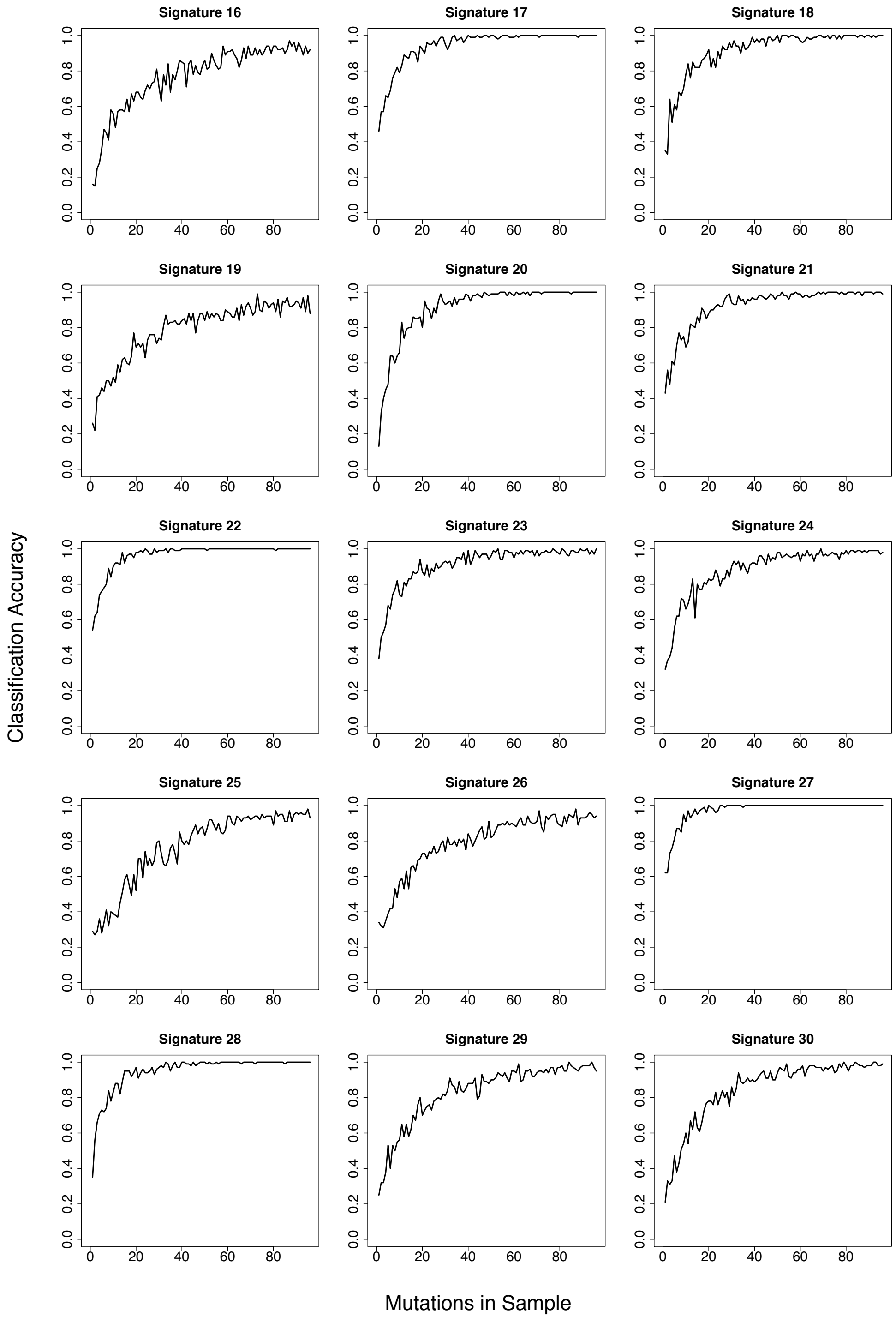

Supplementary Figure 2. Classification accuracies by number of informative mutations for each of signatures 16 to 30 as the true generating signature

Plots show the proportion of 100 simulations where signature assignment was successful (over half of the regression weight assigned to the true generating mutational signature) in simulated mutation data sets.

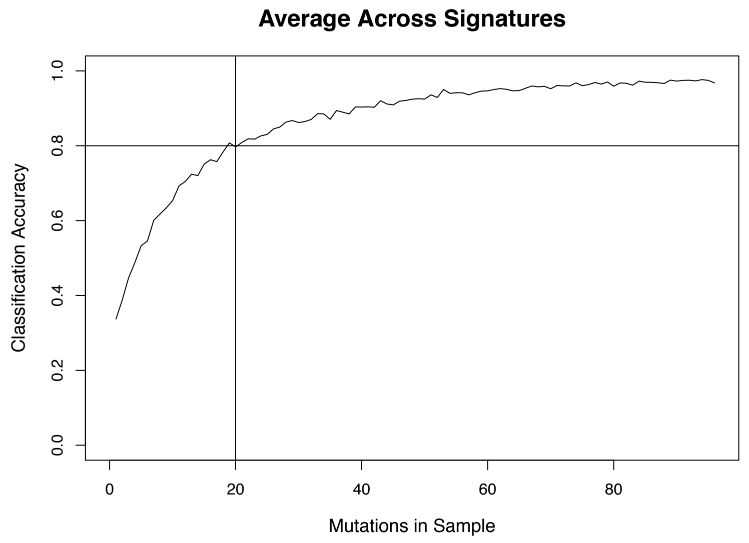

Supplementary Figure 3. Average classification accuracies across 30 mutational signatures

Plot shows average proportion of successful classifications out of 100 simulated data sets across all thirty mutational signatures, based on different numbers of mutations in the simulated data sets.

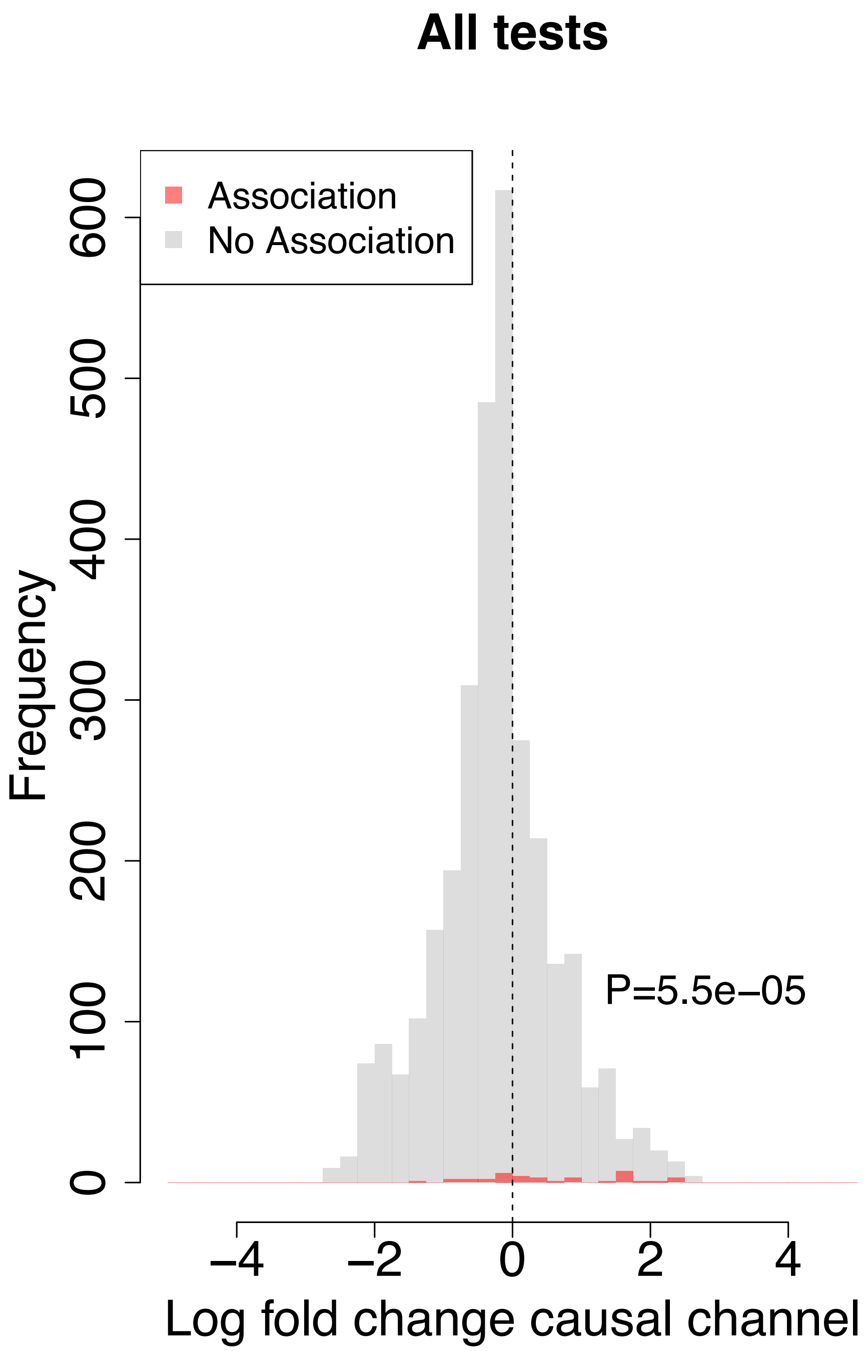

Supplementary Figure 4. Causal channels of associations between mutational signatures and driver mutations

Log fold change of the causal channel of the driver mutation in the mutational signature for significantly associated driver mutations and mutational signatures within cancer types (red) and for those with no association (grey). To calculate the log fold change, 1/96 was added to the probability of the causal channel in the mutational signature, and to the average probability of the causal channel across signatures present in the cancer type. The log fold change shown represents the logarithm of the ratio of the two resulting values.

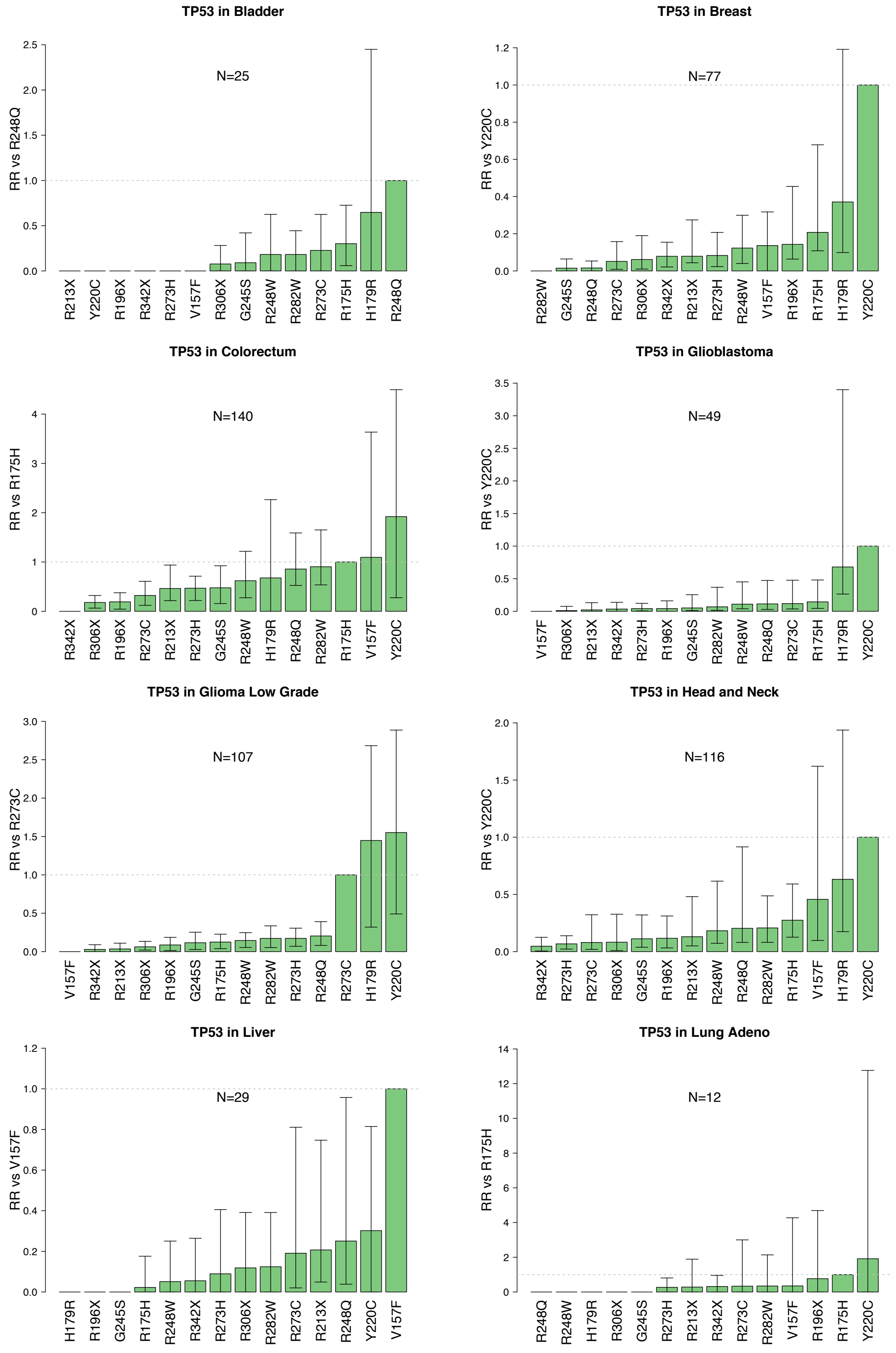

Supplementary Figure 5. Evidence for differential selection between mutations in *TP53* in eight cancer types

Bar plots show modelled relative risk of *TP53* mutations (compared to a reference mutation). Error bars represent 95% confidence intervals obtained by bootstrapping across 100 iterations.

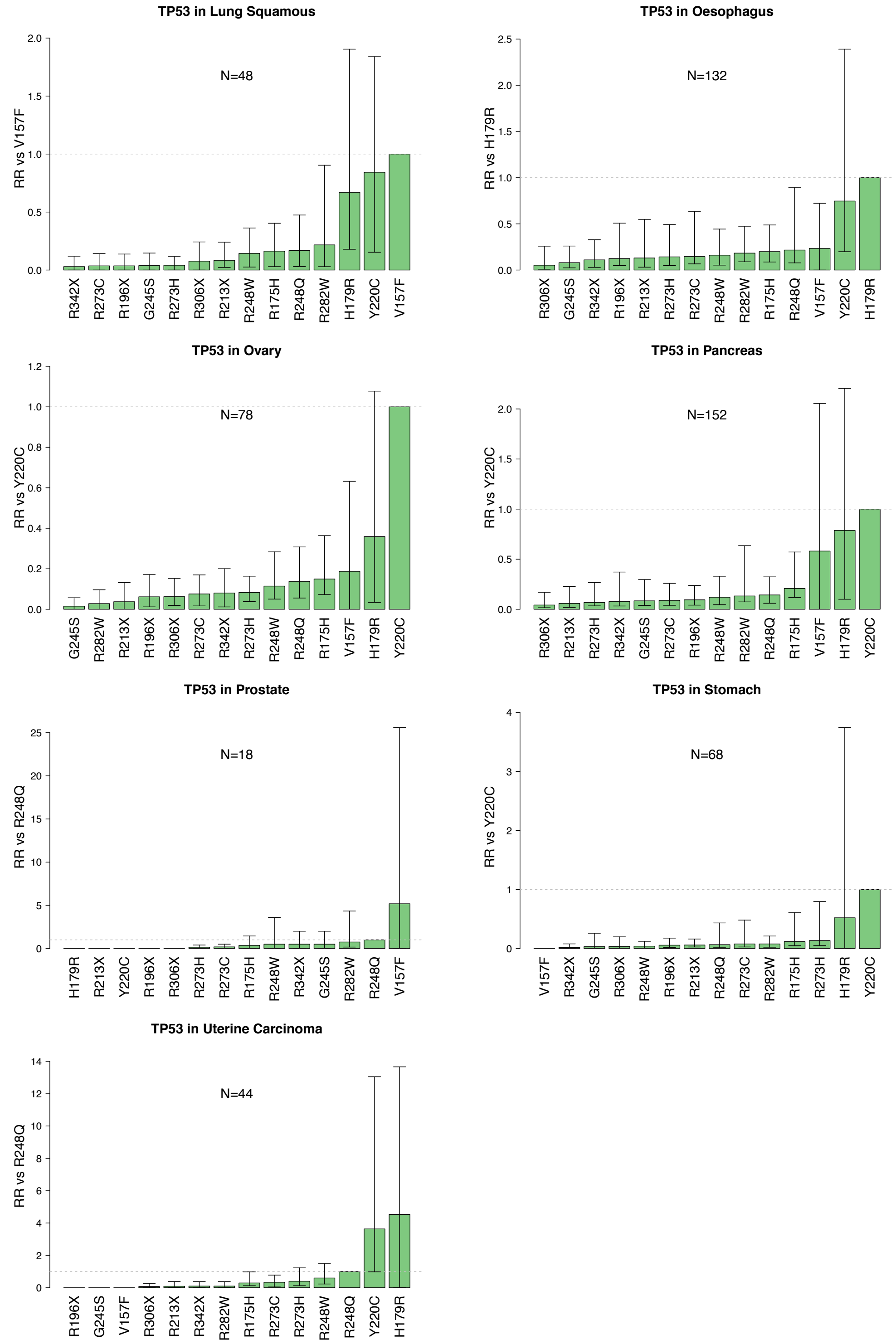

Supplementary Figure 6. Evidence for differential selection between mutations in *TP53* in a further seven cancer types

Bar plots show modelled relative risk of *TP53* mutations (compared to a reference mutation). Error bars represent 95% confidence intervals obtained by bootstrapping across 100 iterations.

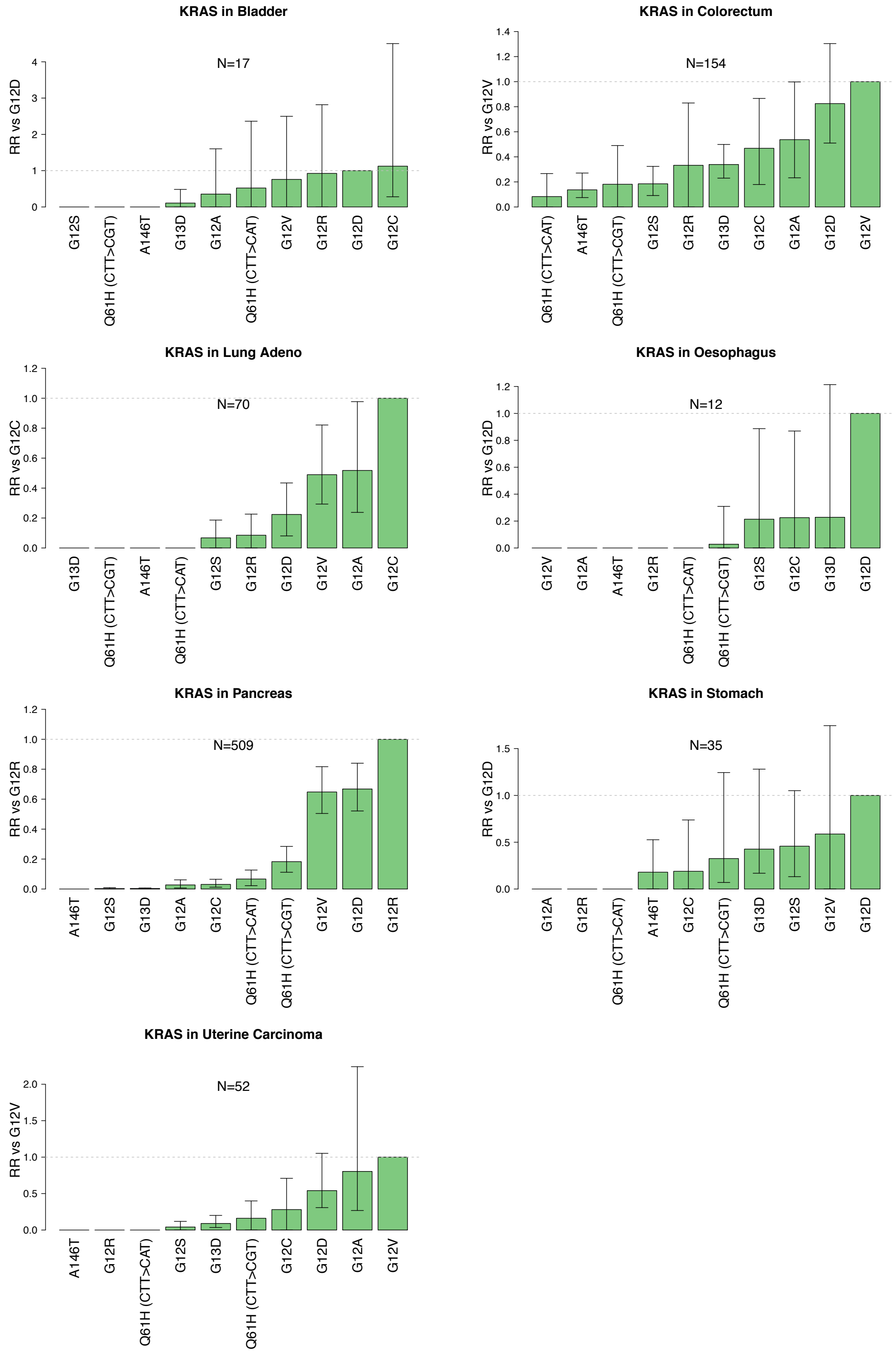

Supplementary Figure 7. Evidence for differential selection between mutations in *KRAS* in seven cancer types

Bar plots show modelled relative risk of *KRAS* mutations (compared to a reference mutation). Error bars represent 95% confidence intervals obtained by bootstrapping across 100 iterations.

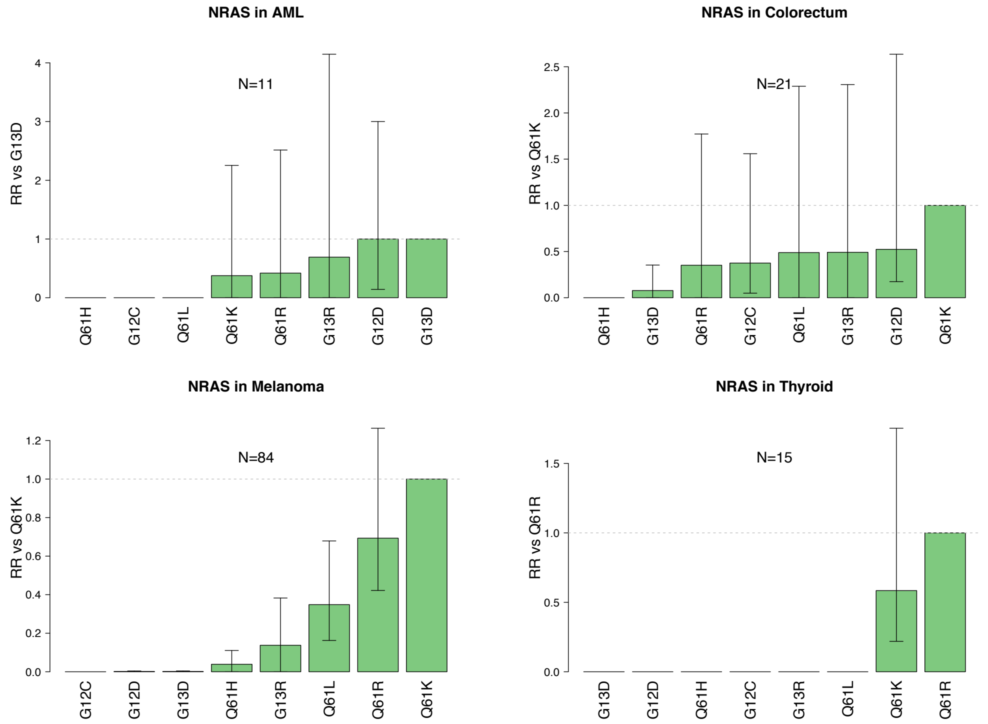

Supplementary Figure 8. Evidence for differential selection between mutations in *NRAS* in four cancer types

Bar plots show modelled relative risk of *NRAS* mutations (compared to a reference mutation). Error bars represent 95% confidence intervals obtained by bootstrapping across 100 iterations.

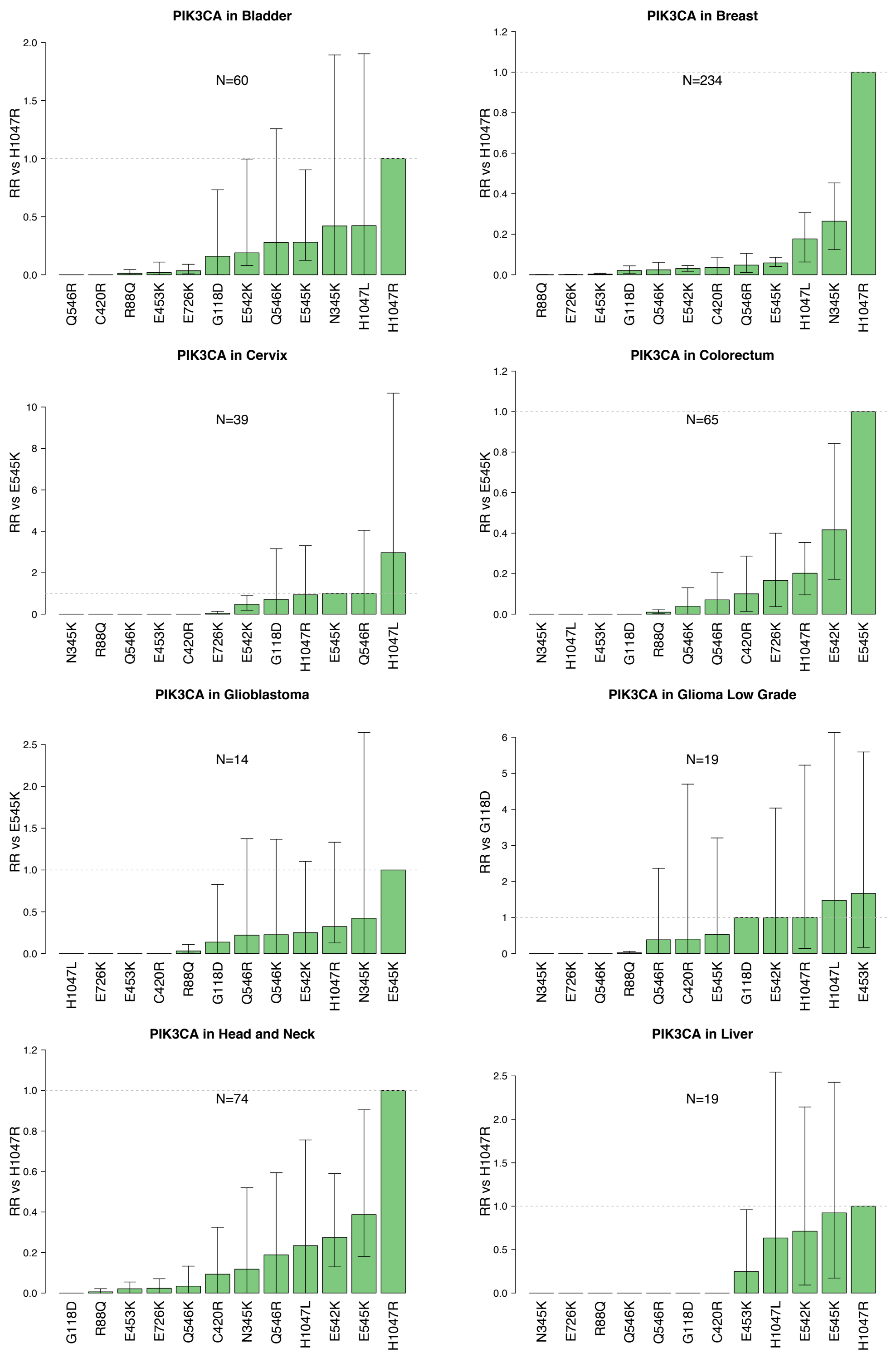

Supplementary Figure 9. Evidence for differential selection between mutations in *PIK3CA* in eight cancer types

Bar plots show modelled relative risk of *PIK3CA* mutations (compared to a reference mutation). Error bars represent 95% confidence intervals obtained by bootstrapping across 100 iterations.

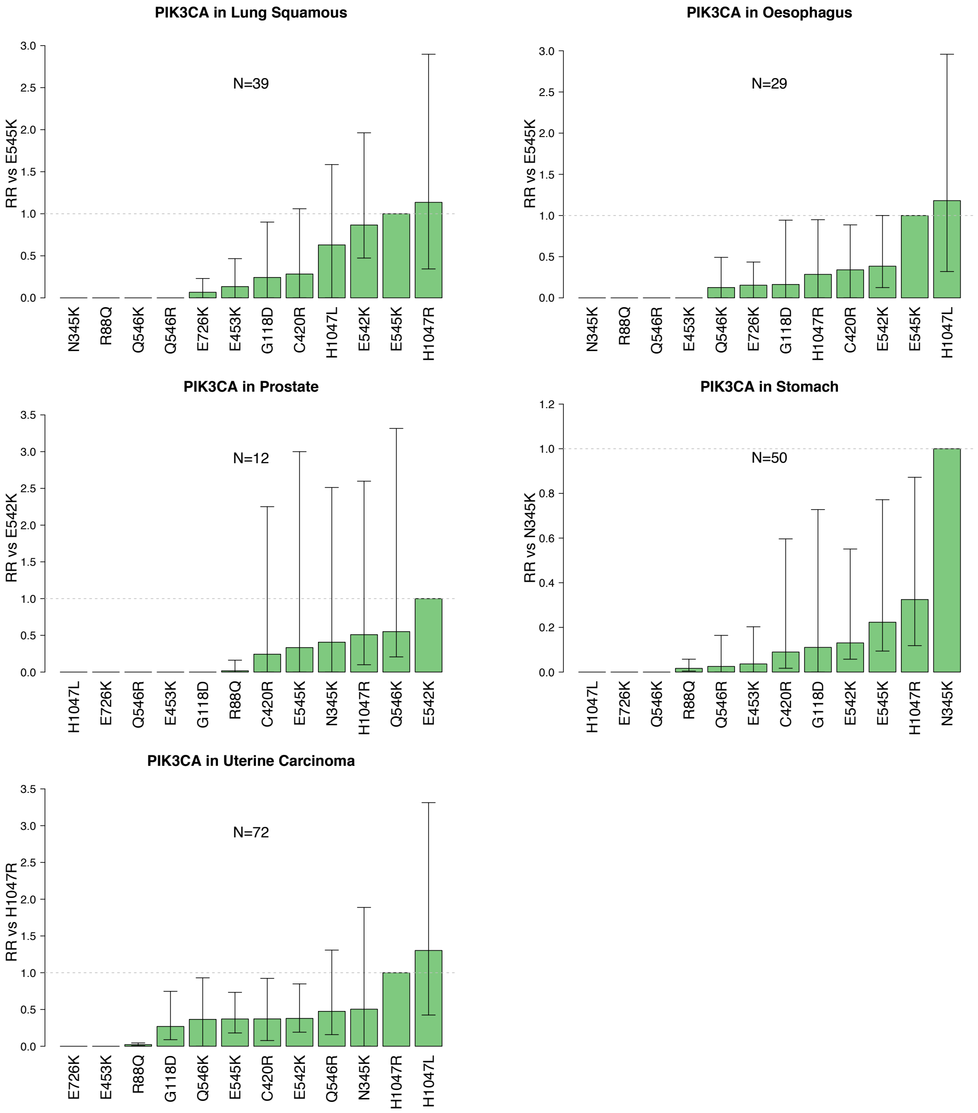

Supplementary Figure 10. Evidence for differential selection between mutations in *PIK3CA* in a further five cancer types

Bar plots show modelled relative risk of *PIK3CA* mutations (compared to a reference mutation). Error bars represent 95% confidence intervals obtained by bootstrapping across 100 iterations.

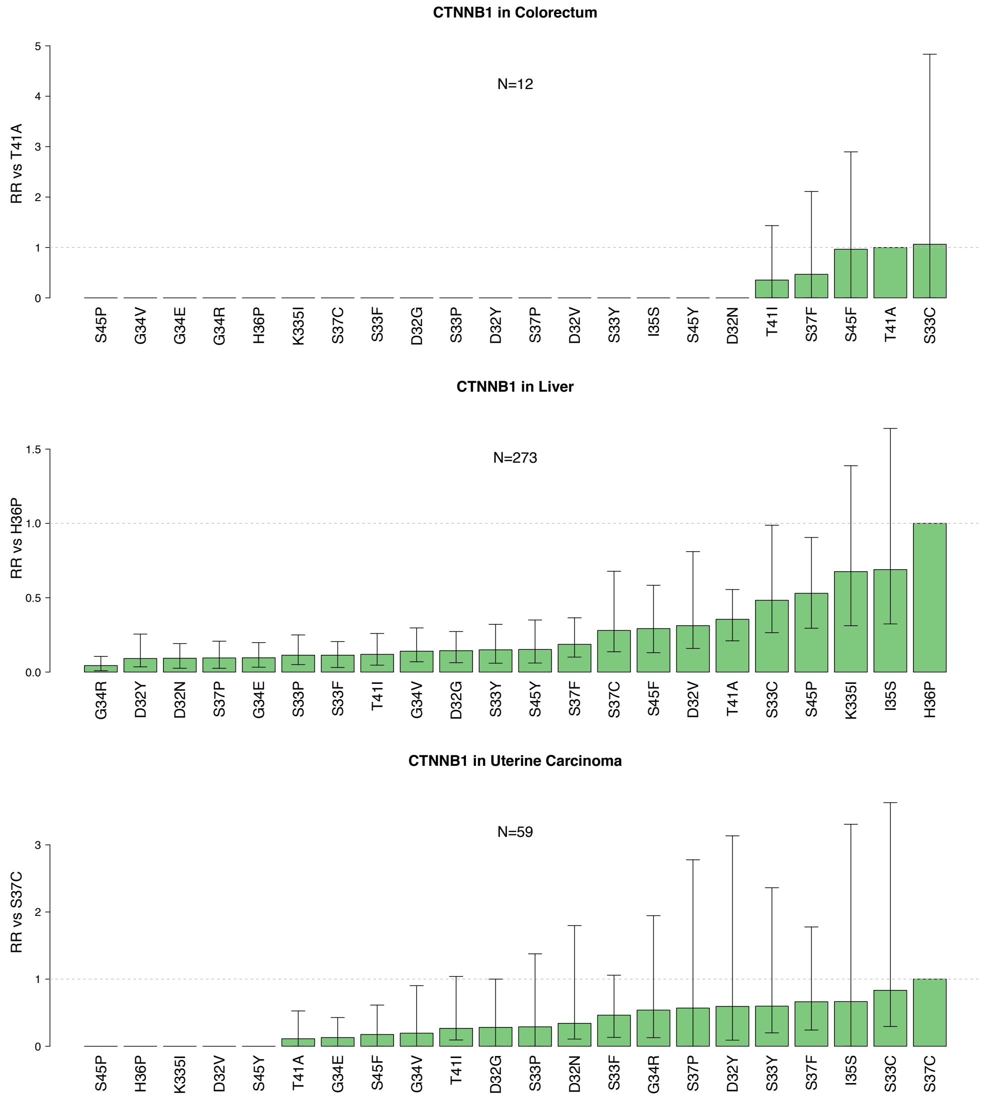

Supplementary Figure 11. Evidence for differential selection between mutations in *CTNNB1* in three cancer types

Bar plots show modelled relative risk of *CTNNB1* mutations (compared to a reference mutation). Error bars represent 95% confidence intervals obtained by bootstrapping across 100 iterations.

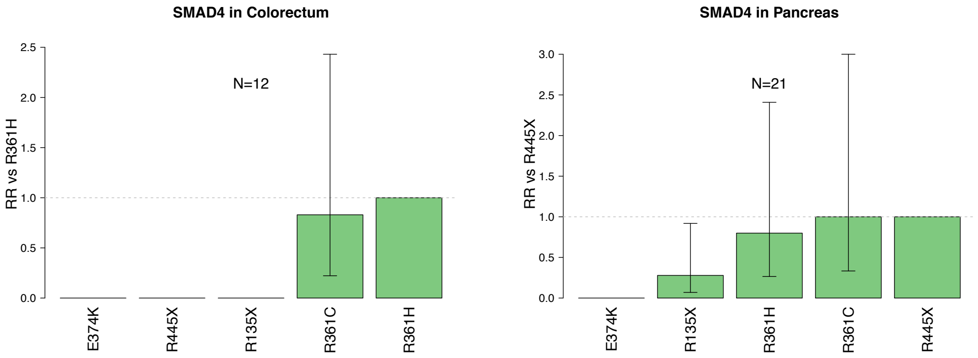

Supplementary Figure 12. Evidence for differential selection between mutations in *SMAD4* in two cancer types

Bar plots show modelled relative risk of *SMAD4* mutations (compared to a reference mutation). Error bars represent 95% confidence intervals obtained by bootstrapping across 100 iterations.

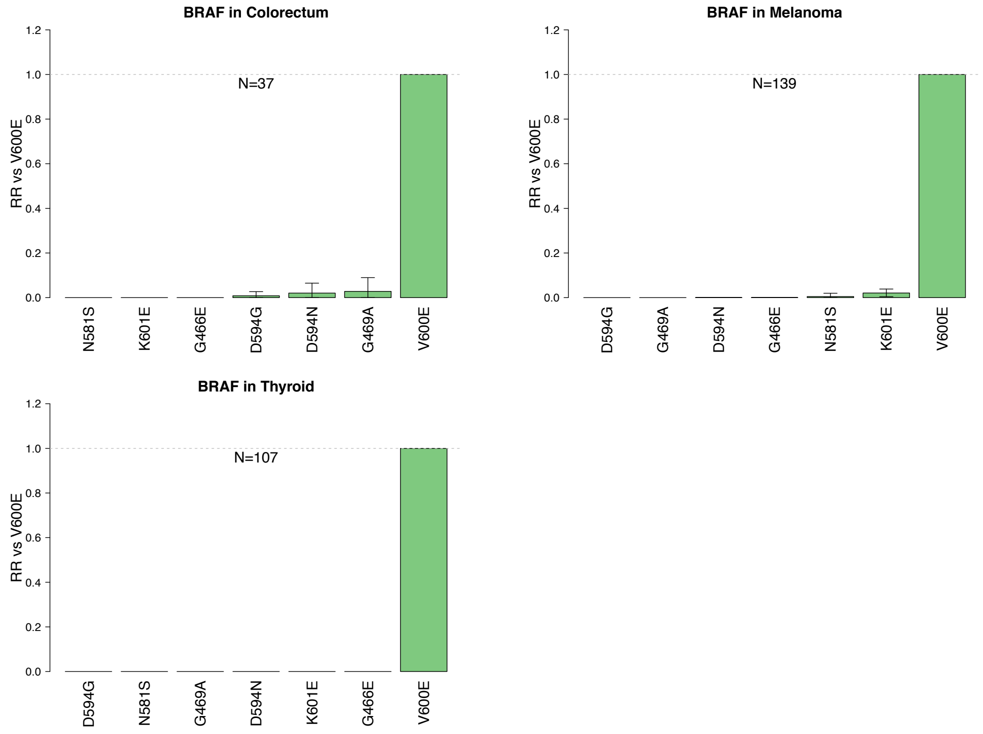

Supplementary Figure 13. Evidence for differential selection between mutations in *BRAF* in three cancer types

Bar plots show modelled relative risk of *BRAF* mutations (compared to a reference mutation). Error bars represent 95% confidence intervals obtained by bootstrapping across 100 iterations.

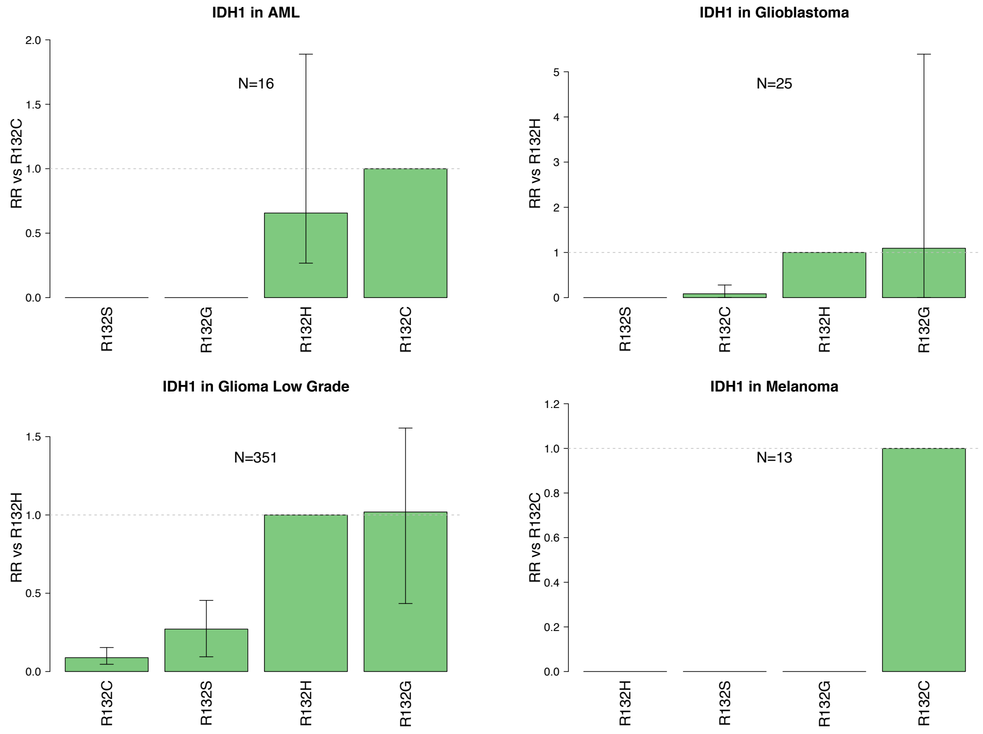

Supplementary Figure 14. Evidence for differential selection between mutations in *IDH1* in four cancer types

Bar plots show modelled relative risk of *IDH1* mutations (compared to a reference mutation). Error bars represent 95% confidence intervals obtained by bootstrapping across 100 iterations.

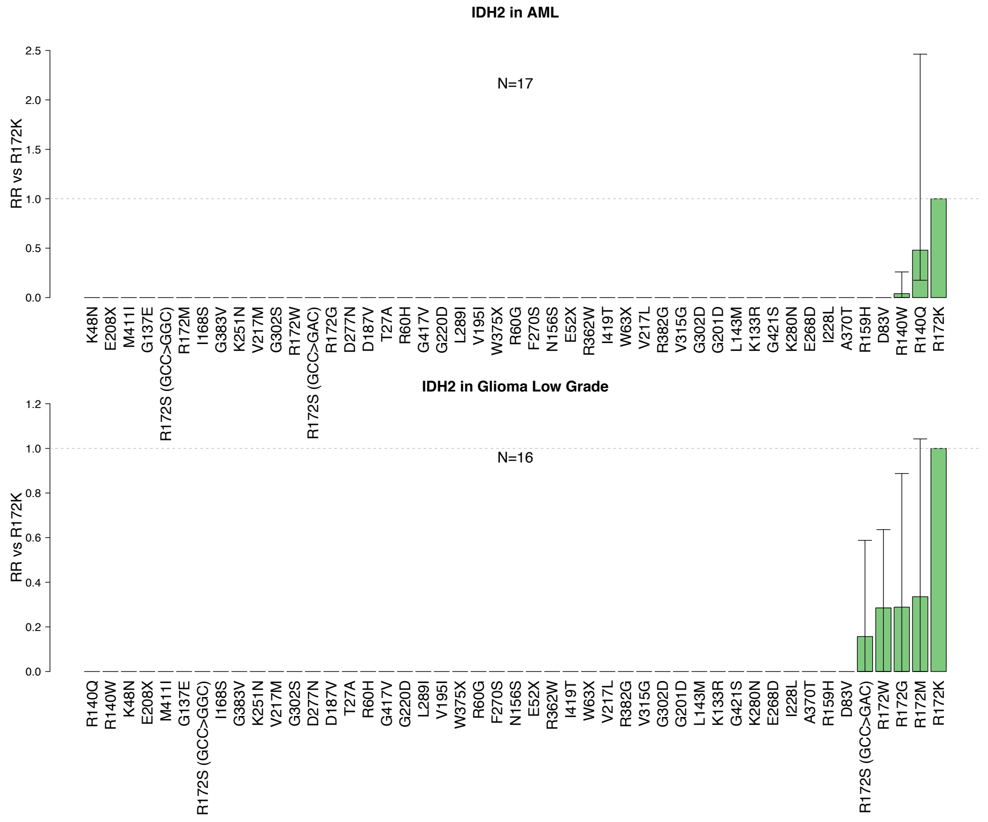

Supplementary Figure 15. Evidence for differential selection between mutations in *IDH2* in two cancer types

Bar plots show modelled relative risk of *IDH2* mutations (compared to a reference mutation). Error bars represent 95% confidence intervals obtained by bootstrapping across 100 iterations.

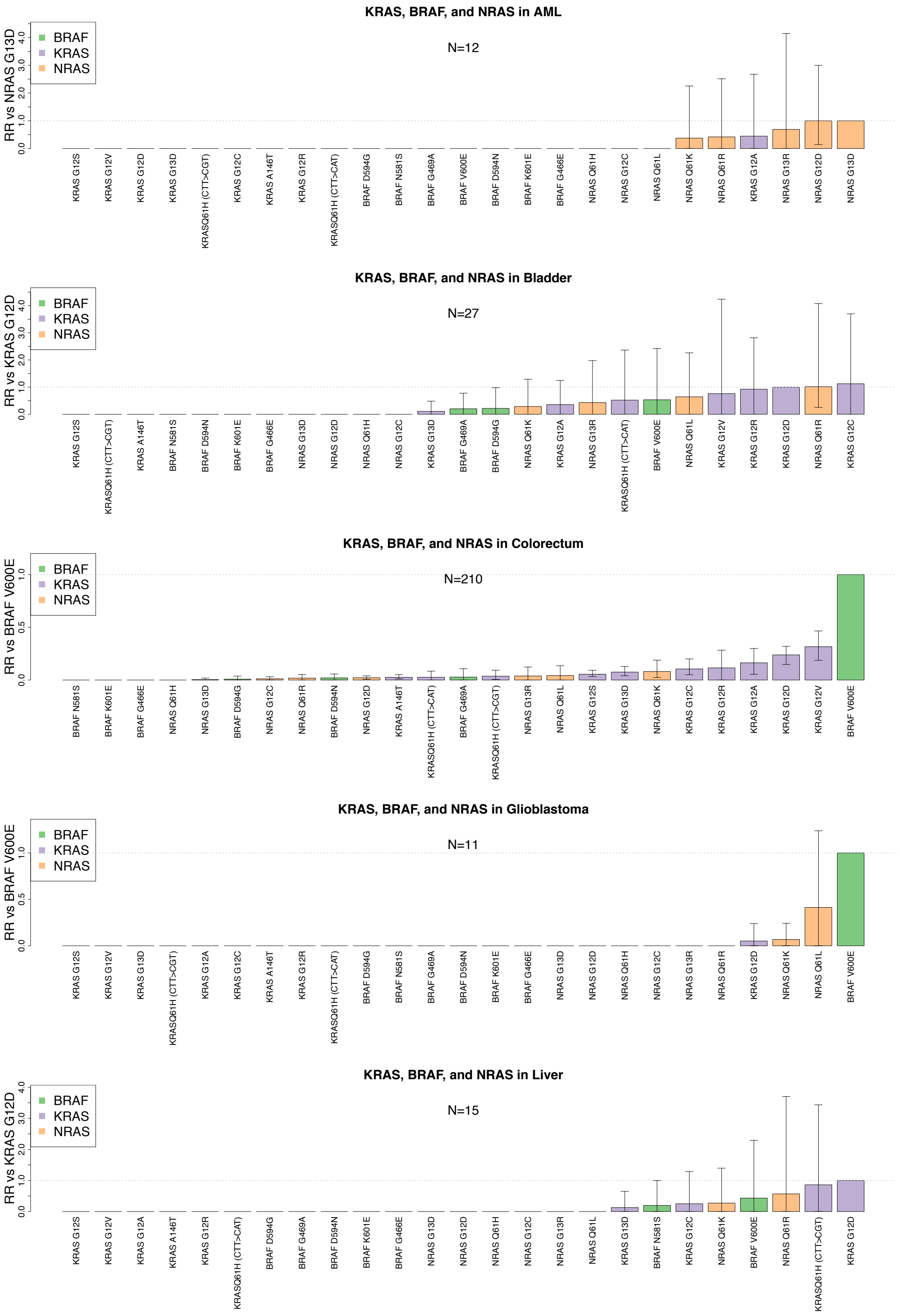

Supplementary Figure 16. Evidence for differential selection between mutations in *KRAS, BRAF,* and *NRAS* in five cancer types

Bar plots show modelled relative risk of *KRAS, BRAF* and *NRAS* mutations (compared to a reference mutation). Error bars represent 95% confidence intervals obtained by bootstrapping across 100 iterations.

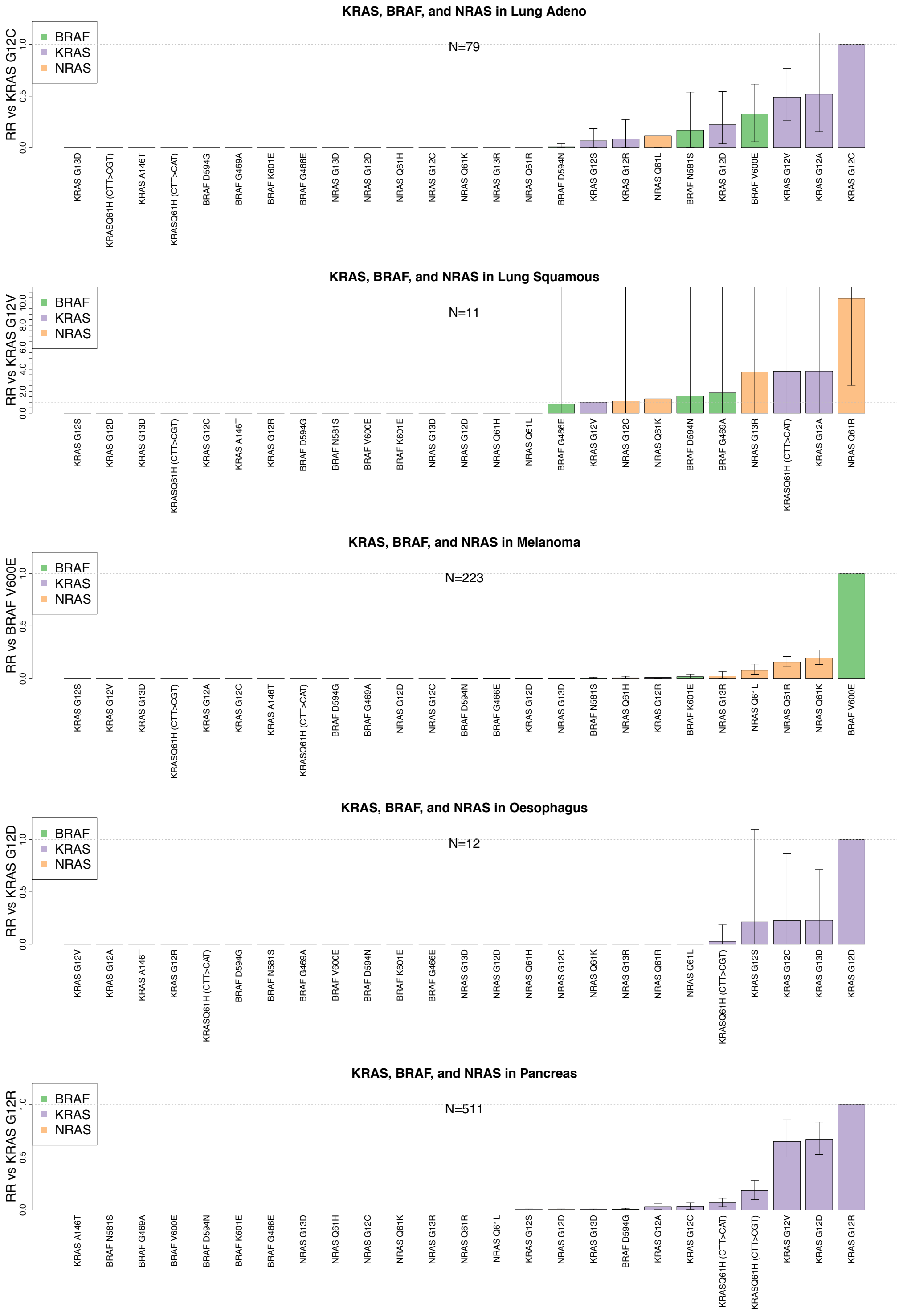

Supplementary Figure 17. Evidence for differential selection between mutations in *KRAS, BRAF,* and *NRAS* in five additional cancer types

Bar plots show modelled relative risk of *KRAS, BRAF* and *NRAS* mutations (compared to a reference mutation). Error bars represent 95% confidence intervals obtained by bootstrapping across 100 iterations. Where the upper confidence interval is not shown in entirety, its value was infinite.

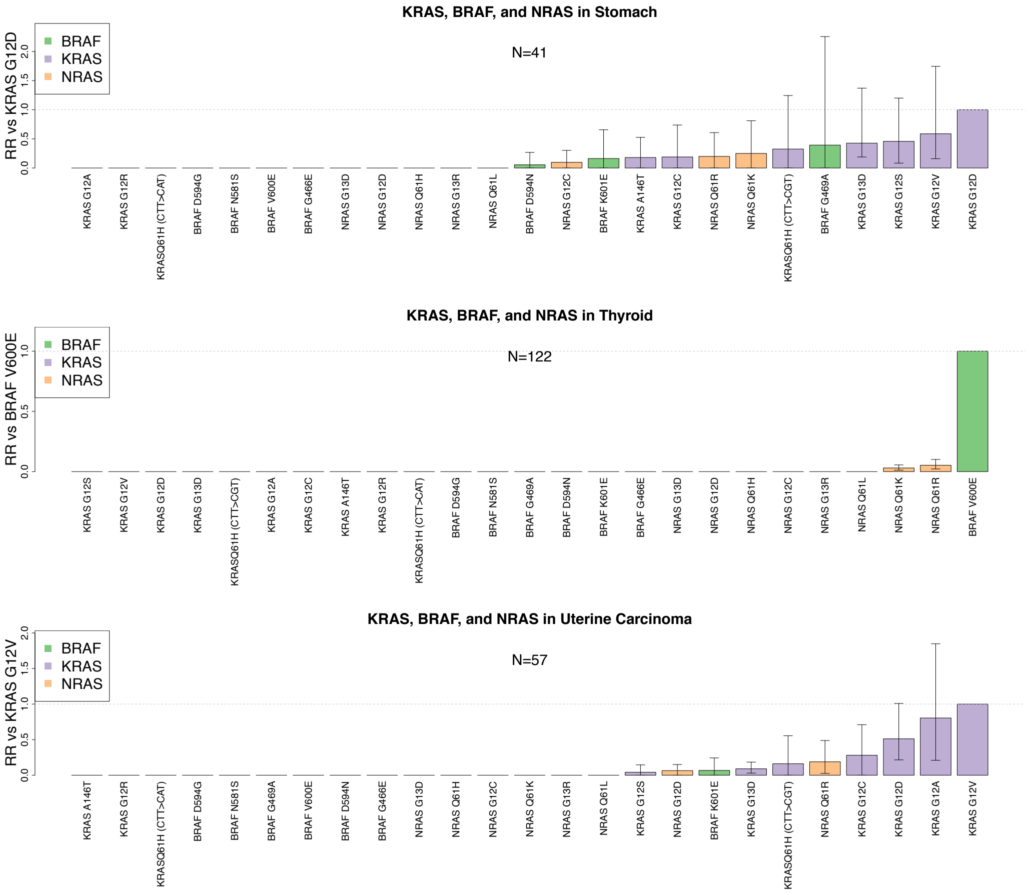

Supplementary Figure 18. Evidence for differential selection between mutations in *KRAS, BRAF,* and *NRAS* in a final three cancer types

Bar plots show modelled relative risk of *KRAS*, *BRAF* and *NRAS* mutations (compared to a reference mutation). Error bars represent 95% confidence intervals obtained by bootstrapping across 100 iterations.

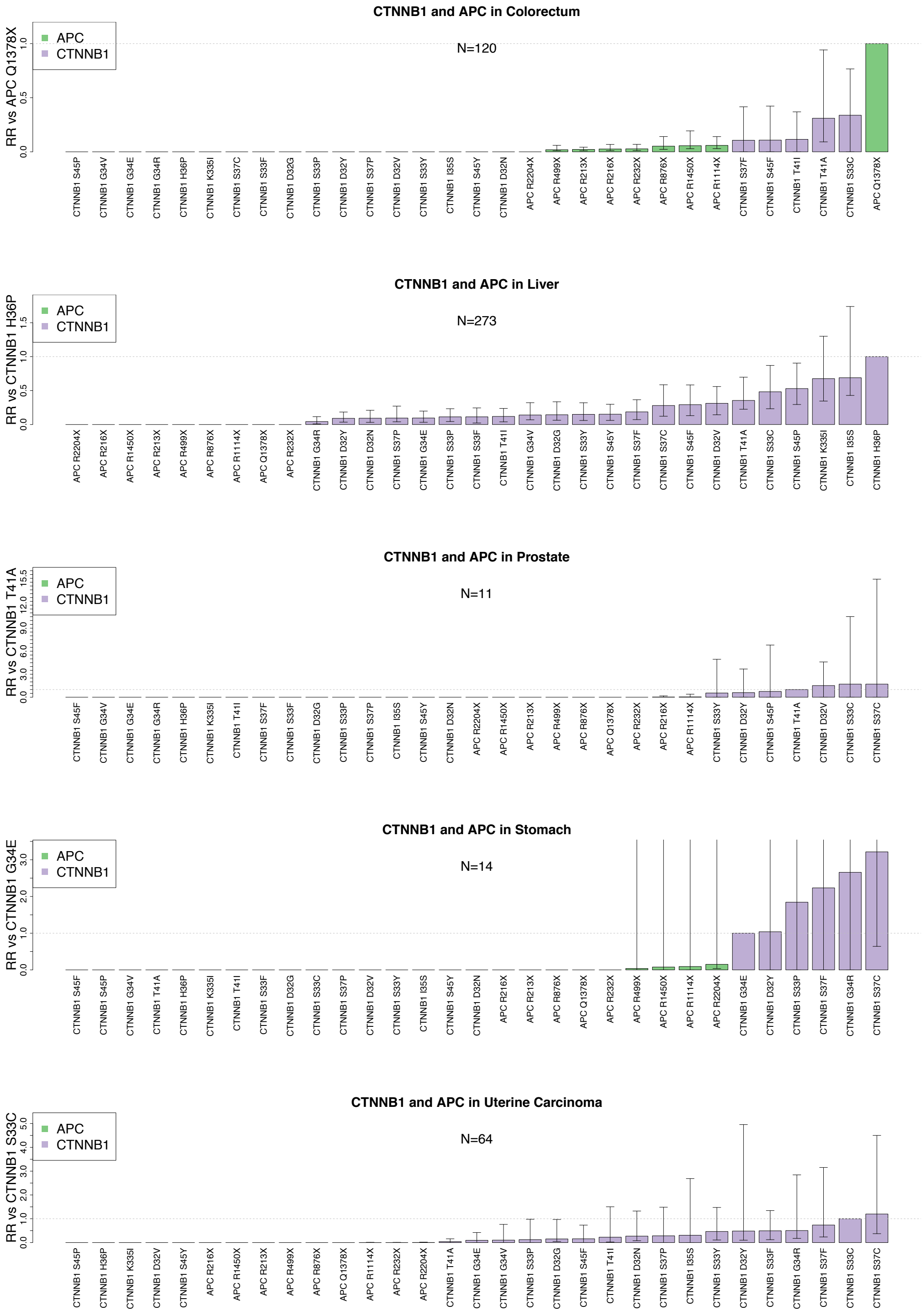

Supplementary Figure 19. Evidence for differential selection between mutations in *APC* and *CTNNB1* in five cancer types

Bar plots show modelled relative risk of *APC* and *CTNNB1* mutations (compared to a reference mutation). Error bars represent 95% confidence intervals obtained by bootstrapping across 100 iterations. Where the upper confidence interval is not shown in entirety, its value was infinite.

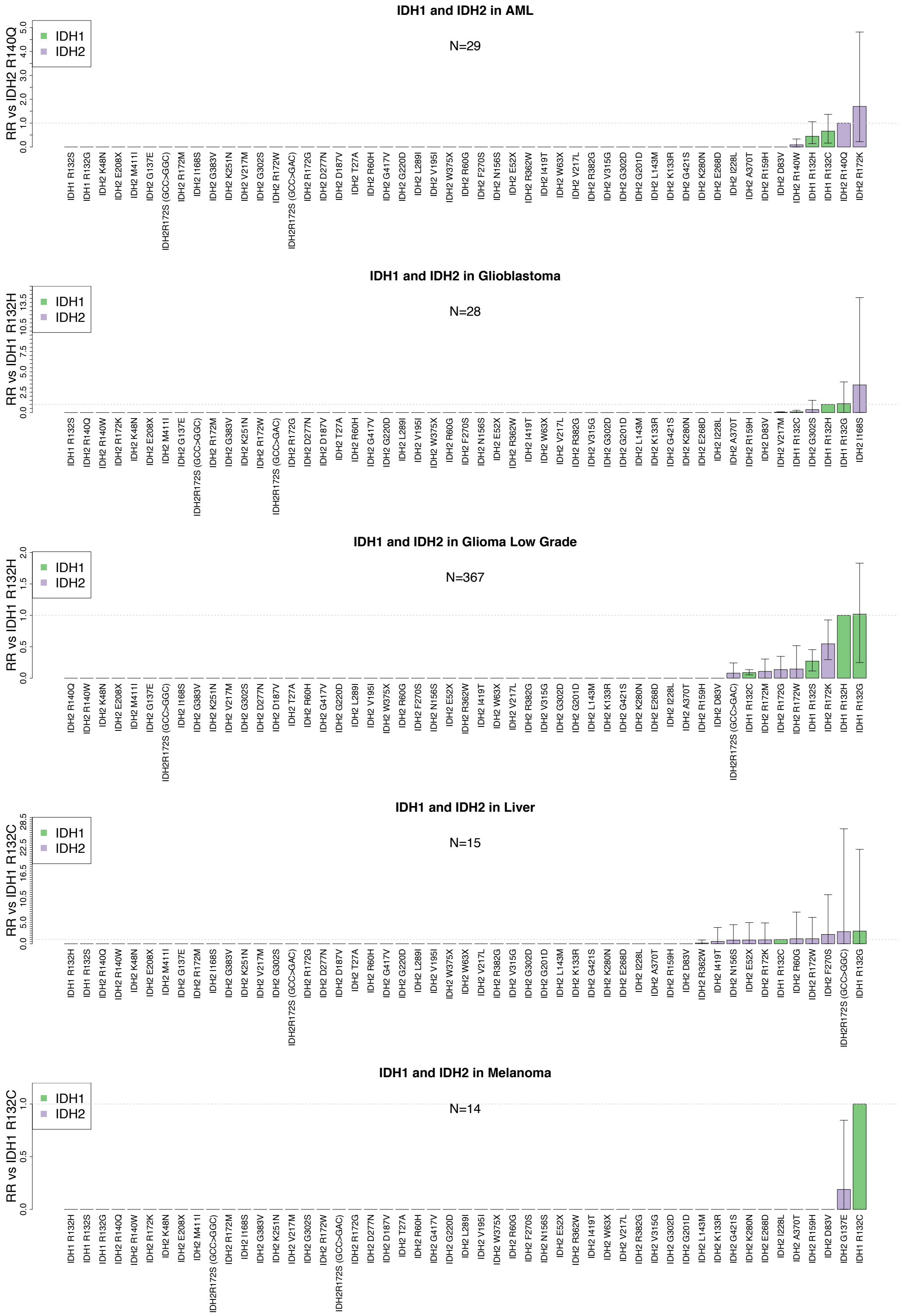

Supplementary Figure 20. Evidence for differential selection between mutations in *IDH1* and *IDH2* in five cancer types

Bar plots show modelled relative risk of *IDH1* and *IDH2* mutations (compared to a reference mutation). Error bars represent 95% confidence intervals obtained by bootstrapping across 100 iterations.

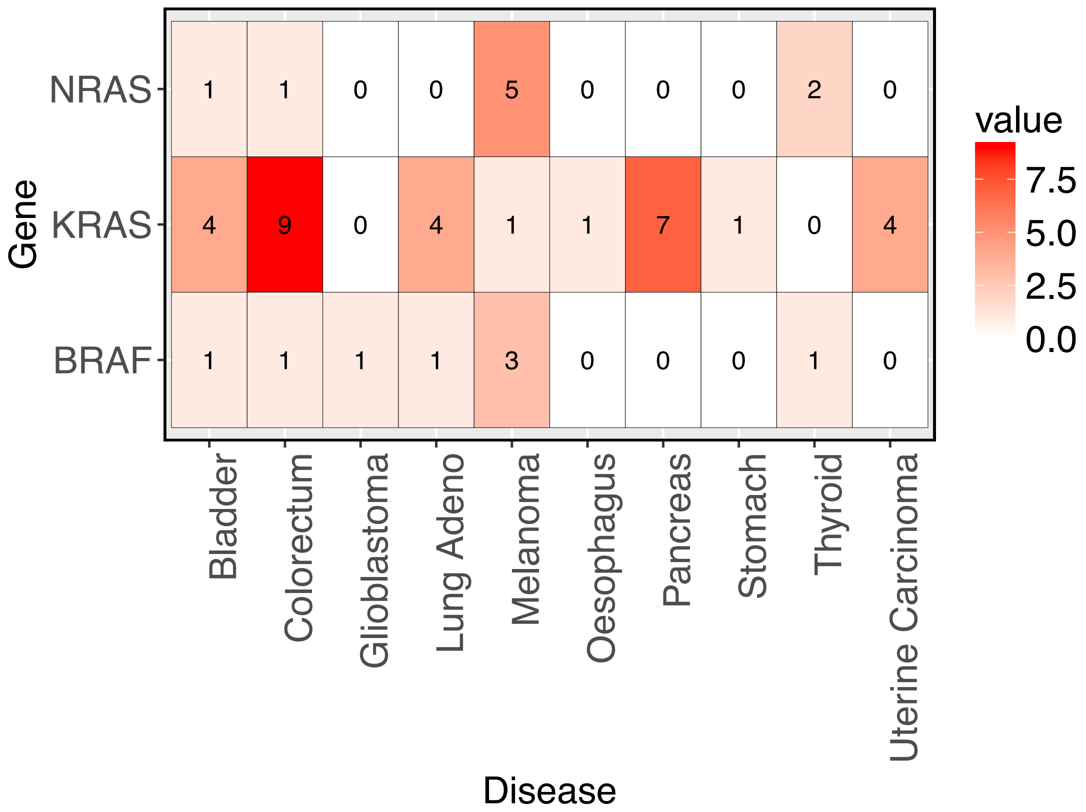

Supplementary Figure 21. The number of mutations from each of KRAS, BRAF and NRAS with a frequency significantly greater than expectation compared to at least one other mutation in each cancer type

The number of mutations showing evidence of preferential selection in each gene showed significant heterogeneity across cancer types. See Supplementary Data 5 for the number of samples with the mutations in question in each cancer type.

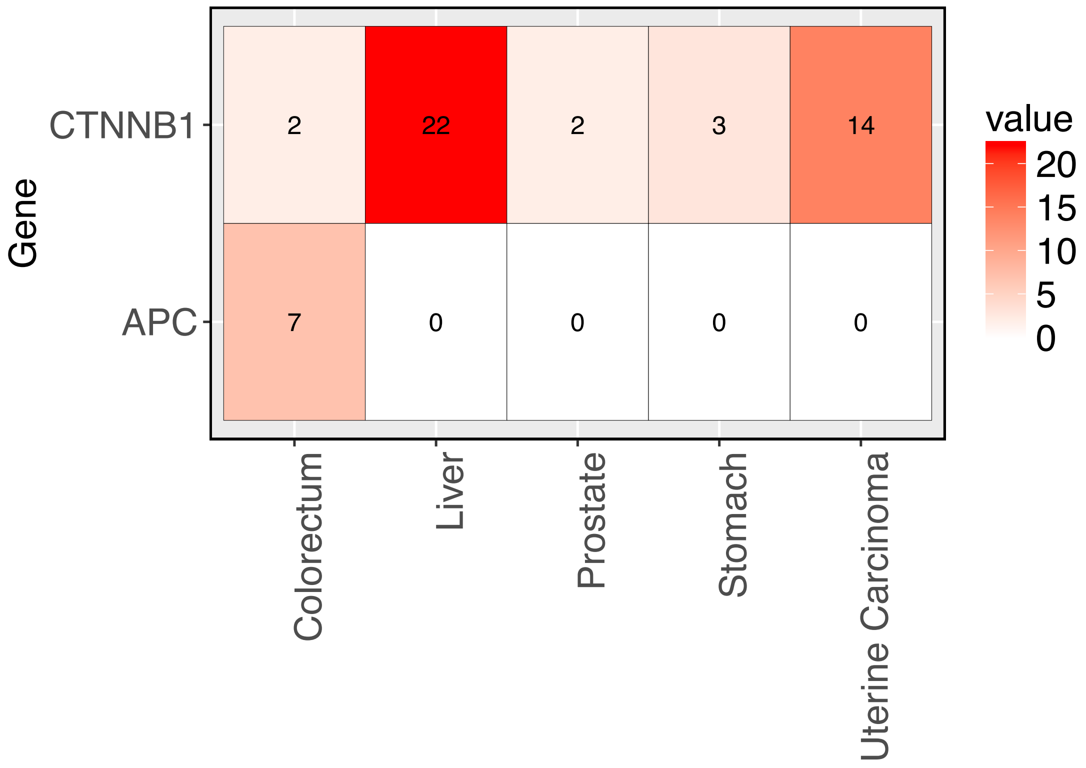

Supplementary Figure 22. The number of mutations from each of CTNNB1 and APC with a frequency significantly greater than expectation compared to at least one other mutation in each cancer type

The number of mutations showing evidence of preferential selection in each gene showed significant heterogeneity across cancer types. See Supplementary Data 5 for the number of samples with the mutations in question in each cancer type.

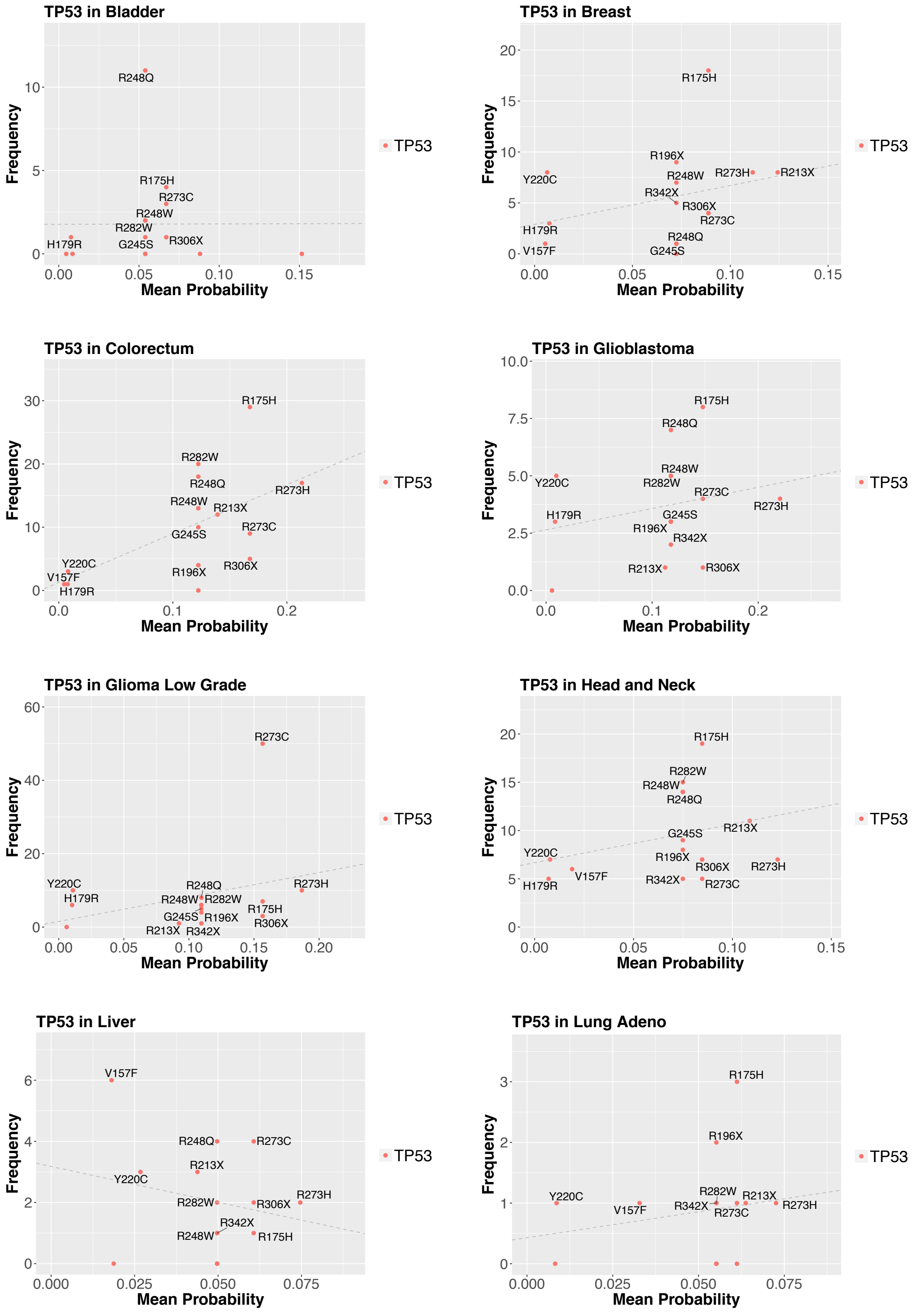

Supplementary Figure 23. Explanation of mutation frequencies by mutation probabilities for *TP53* in eight cancer types

Plots show the number of times each mutation occurred against the mean probability of the mutation. Dotted line shows relationship based on linear regression. See Supplementary Data 4 for the number of samples with the mutations in question in each cancer type.

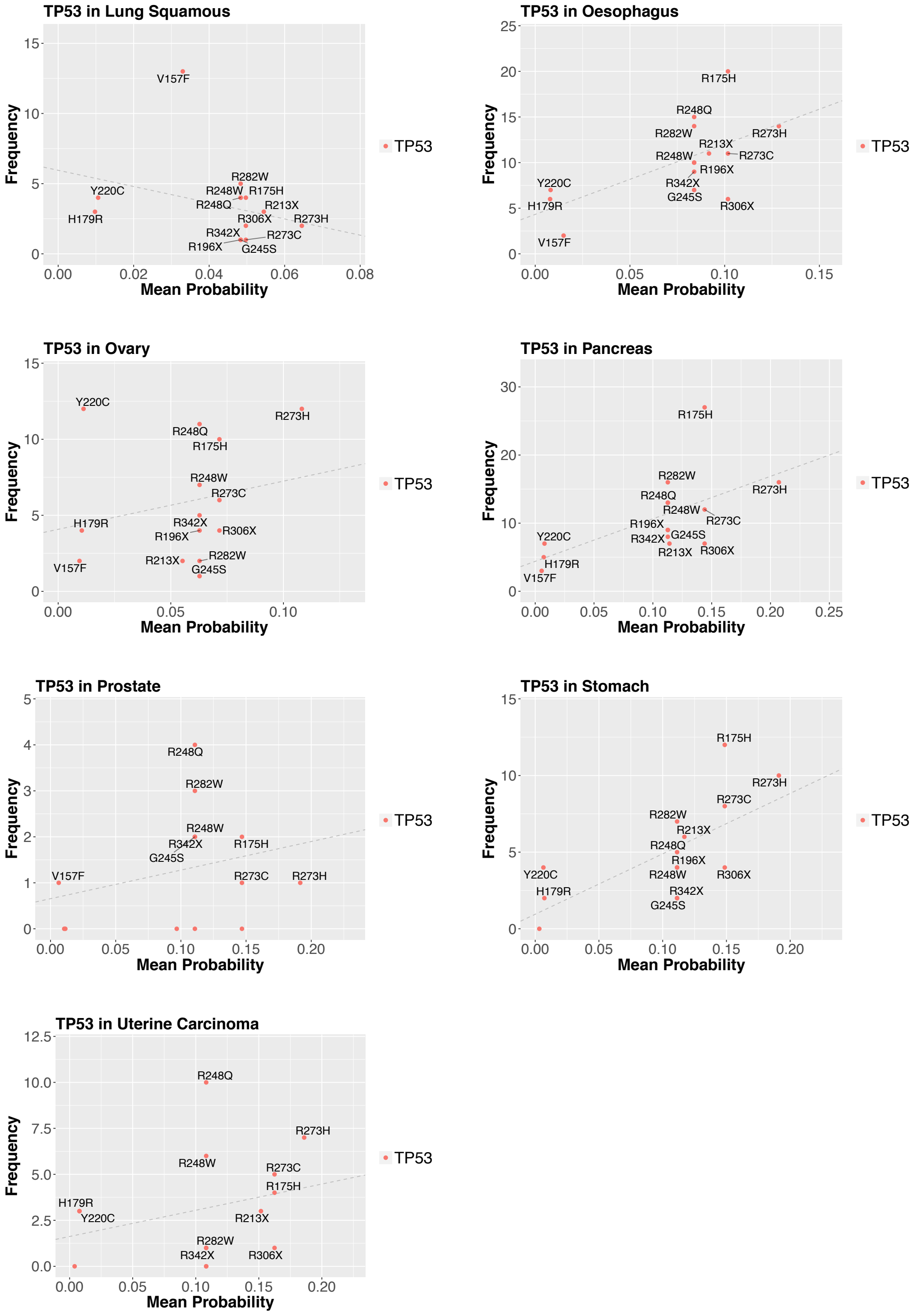

Supplementary Figure 24. Explanation of mutation frequencies by mutation probabilities for *TP53* in a further seven cancer types

Plots show the number of times each mutation occurred against the mean probability of the mutation. Dotted line shows relationship based on linear regression. See Supplementary Data 4 for the number of samples with the mutations in question in each cancer type.

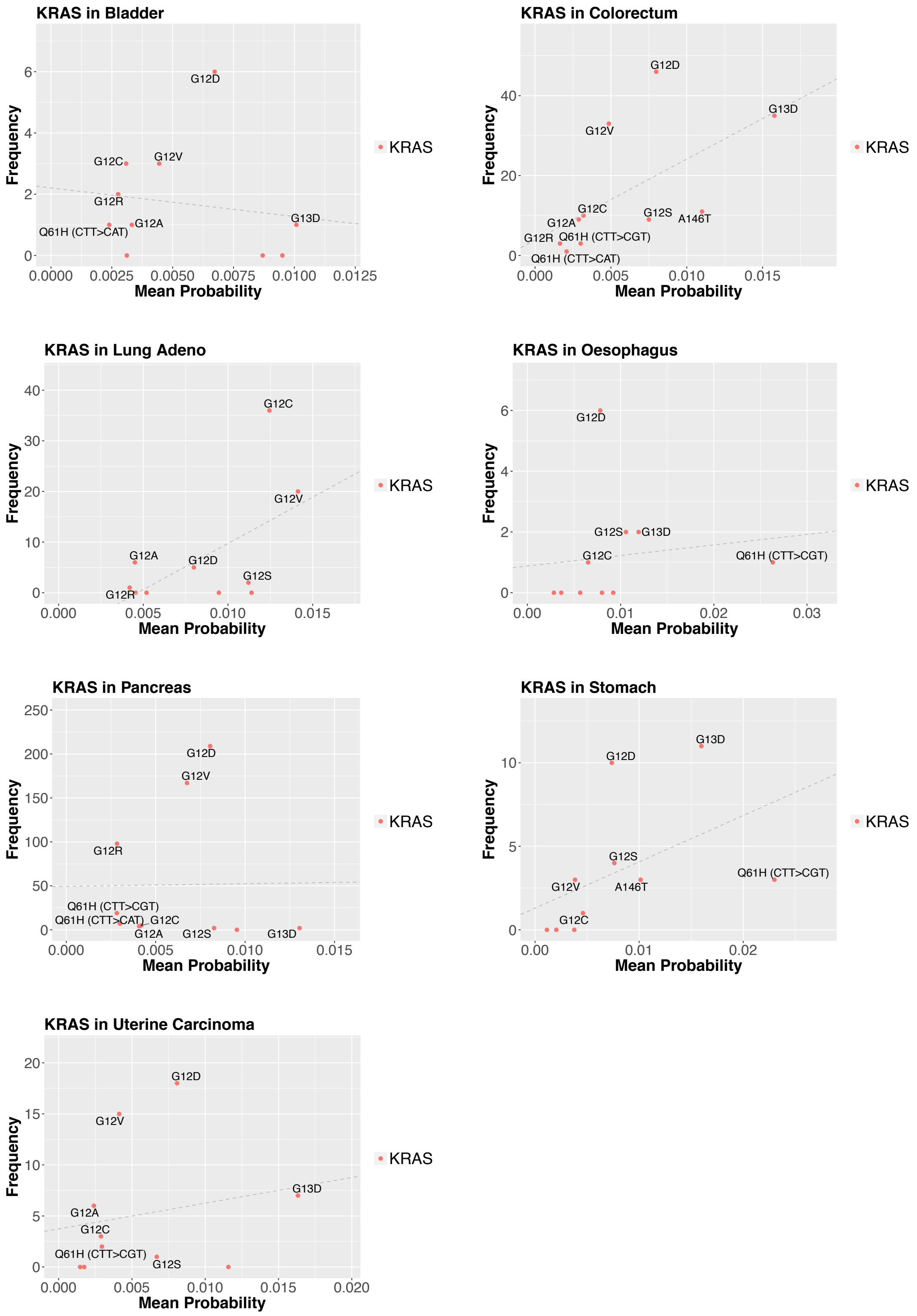

Supplementary Figure 25. Explanation of mutation frequencies by mutation probabilities for *KRAS* in seven cancer types

Plots show the number of times each mutation occurred against the mean probability of the mutation. Dotted line shows relationship based on linear regression. See Supplementary Data 4 for the number of samples with the mutations in question in each cancer type.

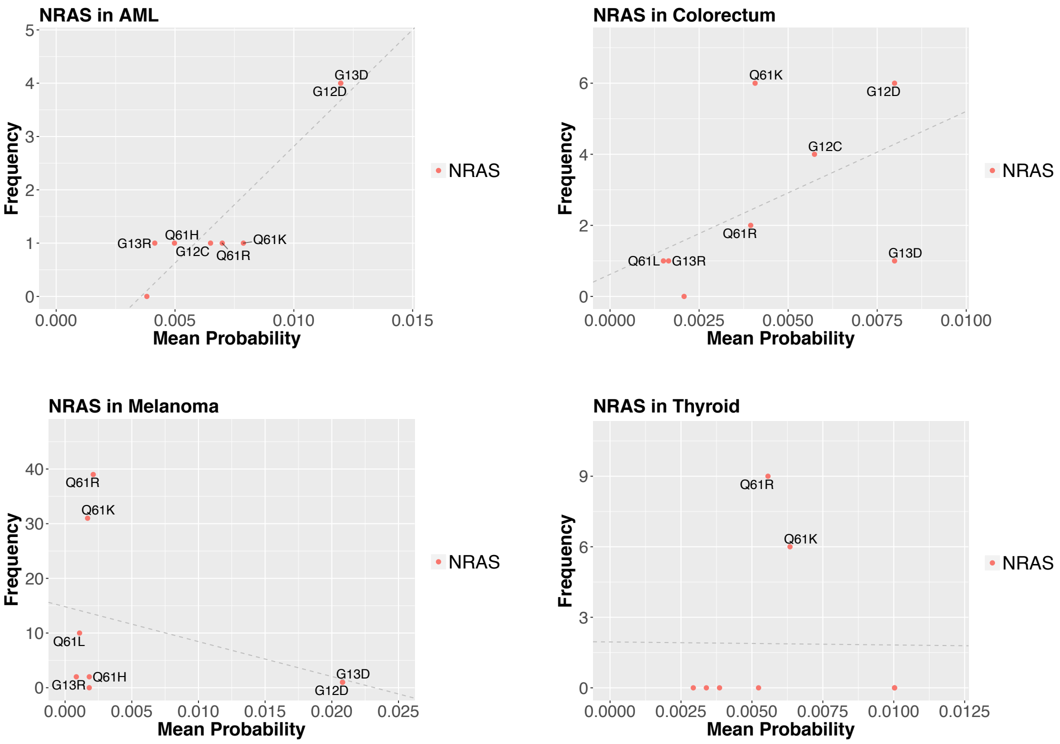

Supplementary Figure 26. Explanation of mutation frequencies by mutation probabilities for *NRAS* in four cancer types

Plots show the number of times each mutation occurred against the mean probability of the mutation. Dotted line shows relationship based on linear regression. See Supplementary Data 4 for the number of samples with the mutations in question in each cancer type.

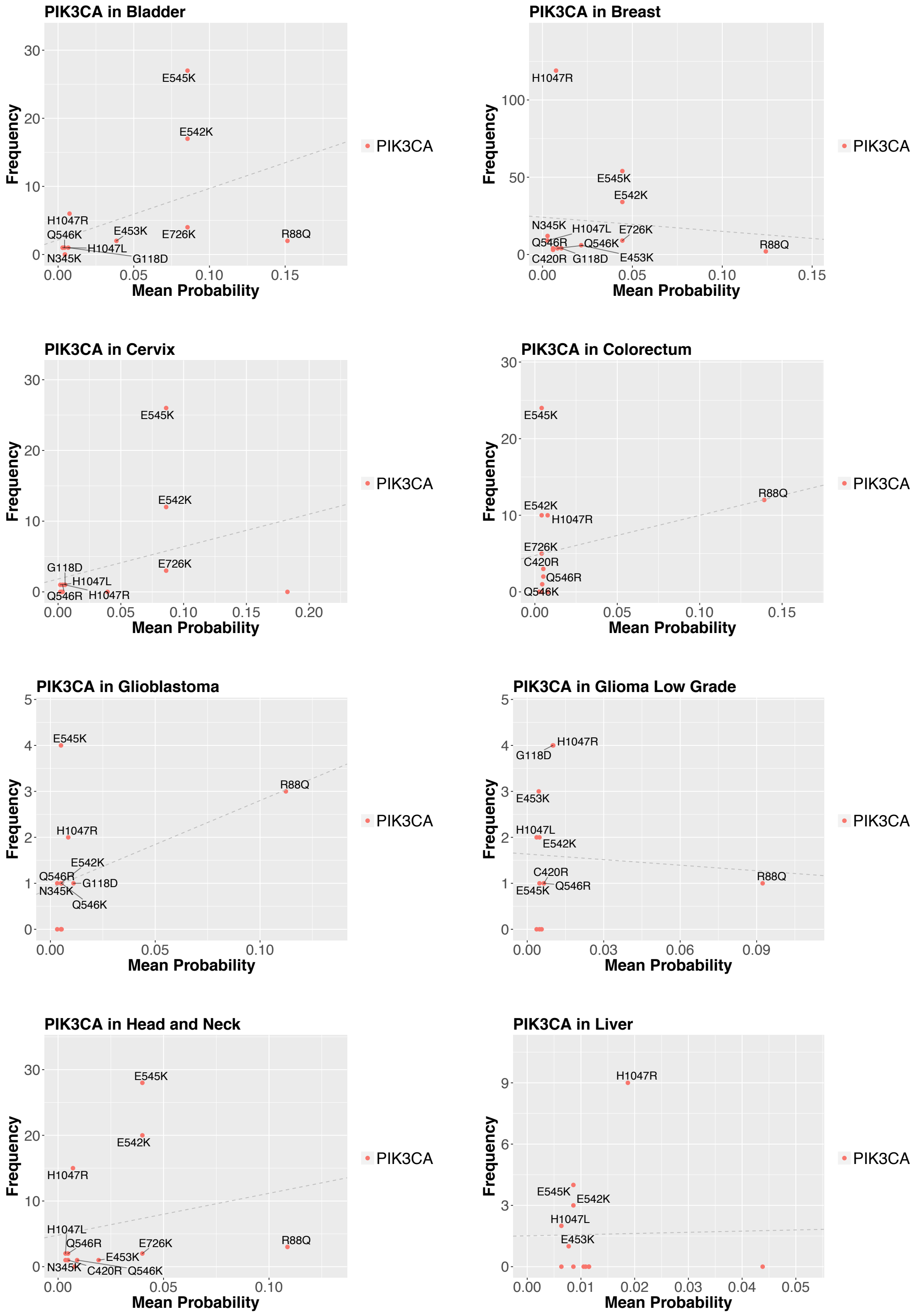

Supplementary Figure 27. Explanation of mutation frequencies by mutation probabilities for *PIK3CA* in eight cancer types

Plots show the number of times each mutation occurred against the mean probability of the mutation. Dotted line shows relationship based on linear regression. See Supplementary Data 4 for the number of samples with the mutations in question in each cancer type.

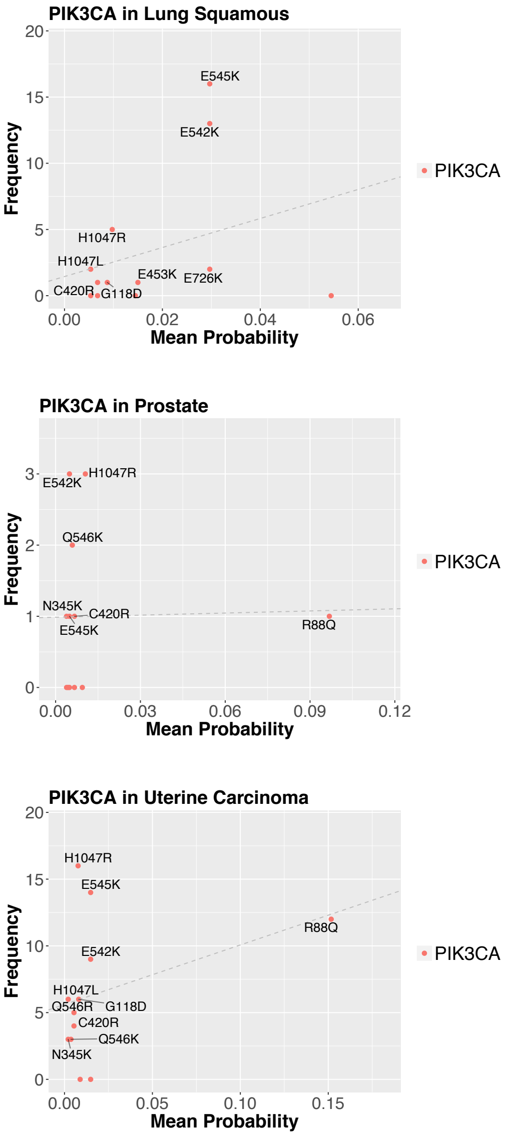

Supplementary Figure 28. Explanation of mutation frequencies by mutation probabilities for *PIK3CA* in a further five cancer types

Plots show the number of times each mutation occurred against the mean probability of the mutation. Dotted line shows relationship based on linear regression. See Supplementary Data 4 for the number of samples with the mutations in question in each cancer type.

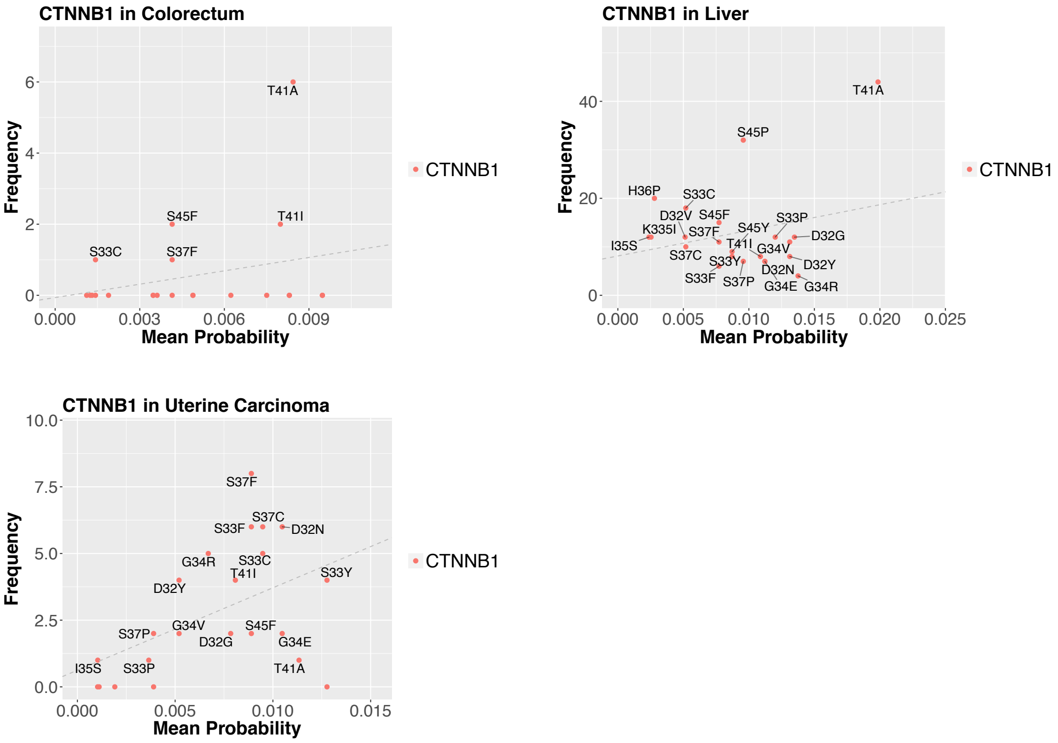

Supplementary Figure 29. Explanation of mutation frequencies by mutation probabilities for *CTNNB1* in three cancer types

Plots show the number of times each mutation occurred against the mean probability of the mutation. Dotted line shows relationship based on linear regression. See Supplementary Data 4 for the number of samples with the mutations in question in each cancer type.

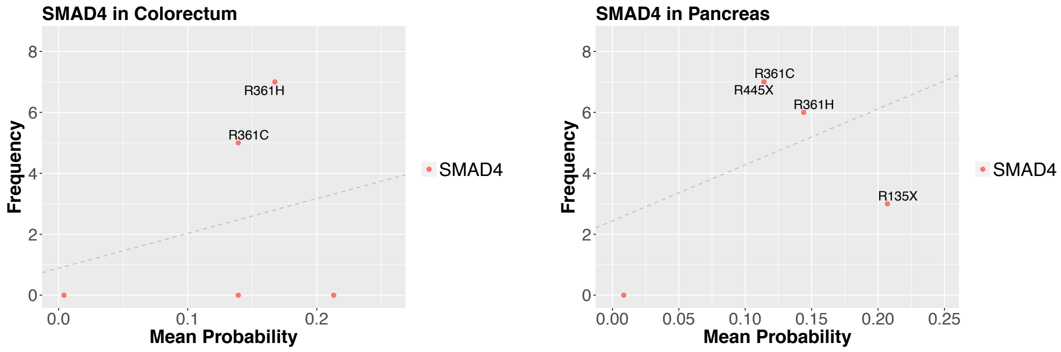

Supplementary Figure 30. Explanation of mutation frequencies by mutation probabilities for *SMAD4* in two cancer types

Plots show the number of times each mutation occurred against the mean probability of the mutation. Dotted line shows relationship based on linear regression. See Supplementary Data 4 for the number of samples with the mutations in question in each cancer type.

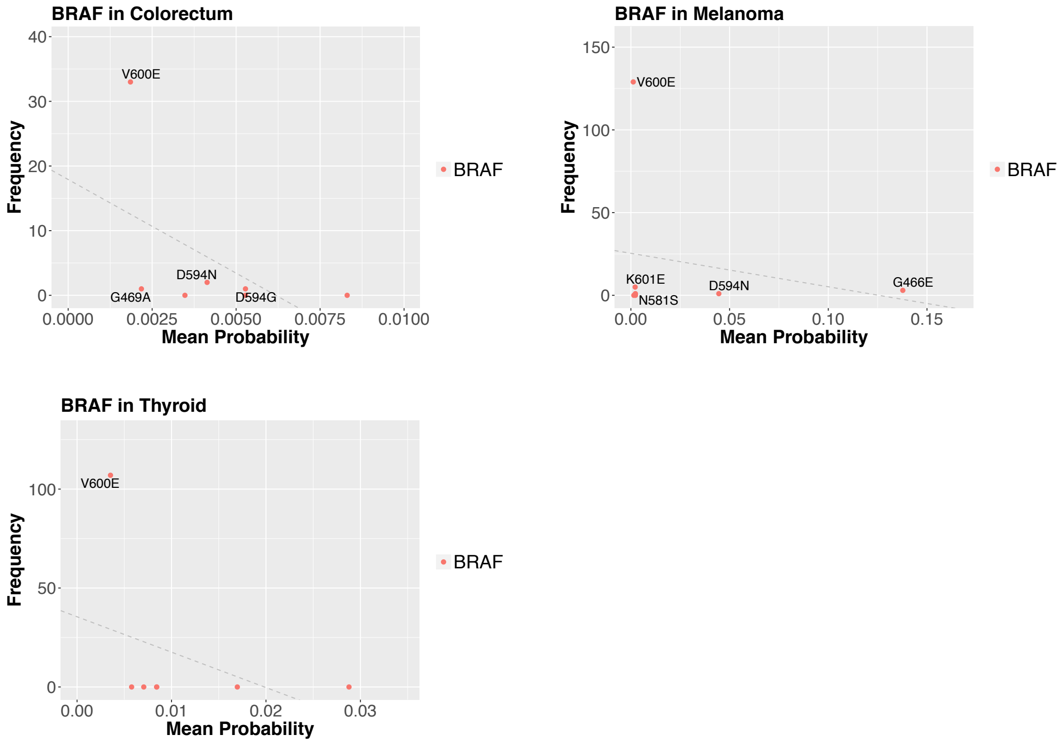

Supplementary Figure 31. Explanation of mutation frequencies by mutation probabilities for *BRAF* in three cancer types

Plots show the number of times each mutation occurred against the mean probability of the mutation. Dotted line shows relationship based on linear regression. See Supplementary Data 4 for the number of samples with the mutations in question in each cancer type.

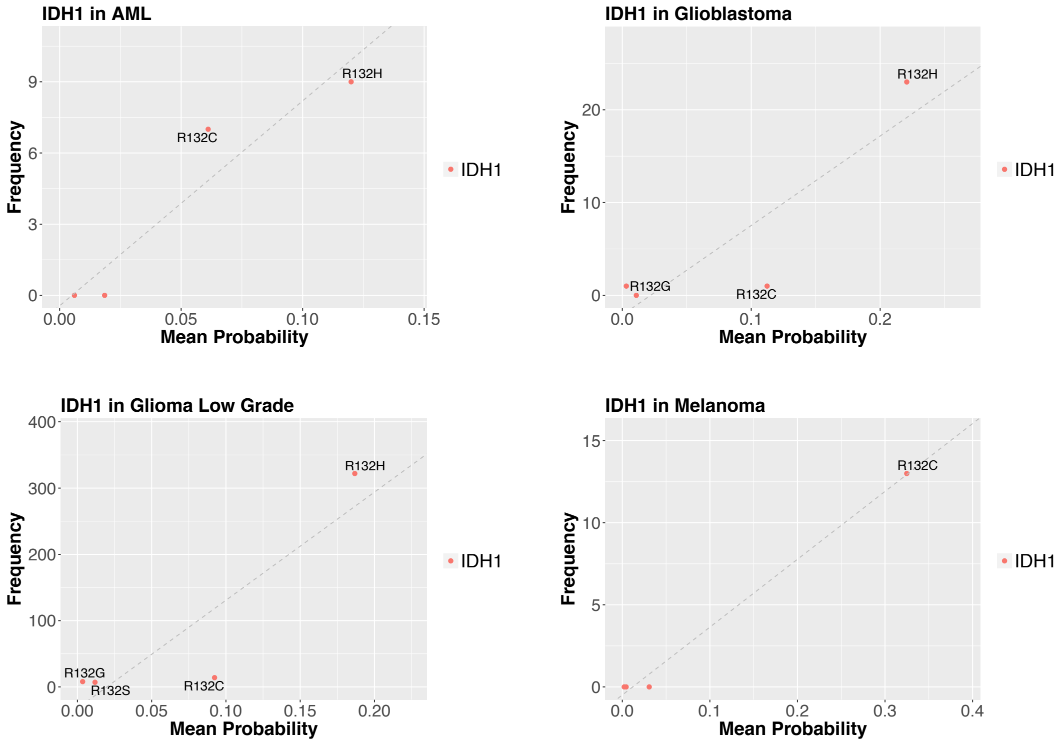

Supplementary Figure 32. Explanation of mutation frequencies by mutation probabilities for *IDH1* in four cancer types

Plots show the number of times each mutation occurred against the mean probability of the mutation. Dotted line shows relationship based on linear regression. See Supplementary Data 4 for the number of samples with the mutations in question in each cancer type.

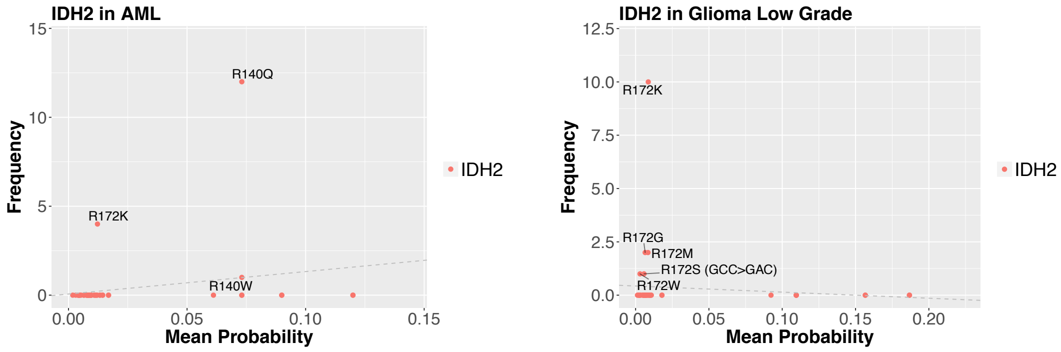

Supplementary Figure 33. Explanation of mutation frequencies by mutation probabilities for *IDH2* in two cancer types

Plots show the number of times each mutation occurred against the mean probability of the mutation. Dotted line shows relationship based on linear regression. See Supplementary Data 4 for the number of samples with the mutations in question in each cancer type.

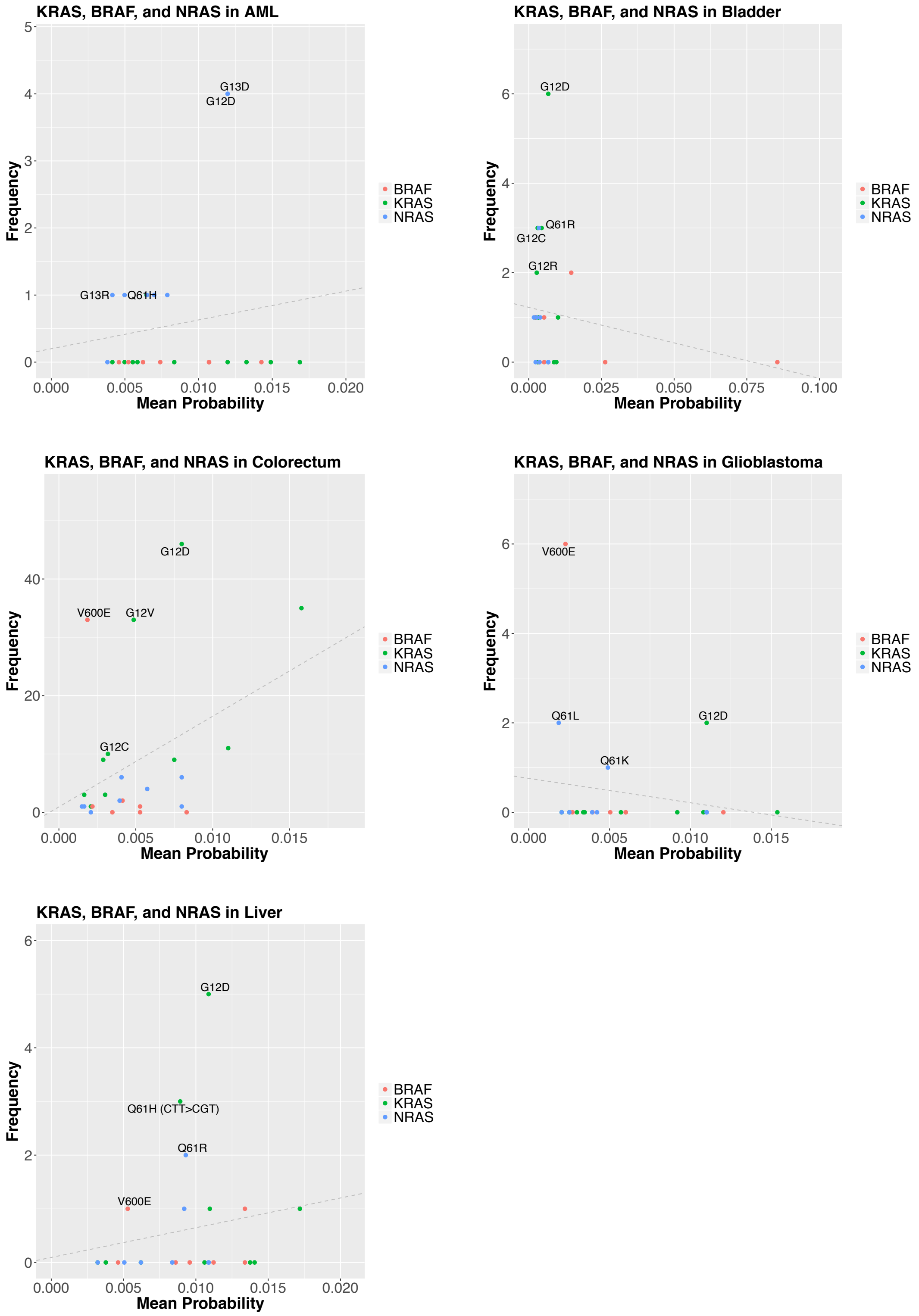

Supplementary Figure 34. Explanation of mutation frequencies by mutation probabilities for *BRAF, KRAS,* and *NRAS* in five cancer types

Plots show the number of times each mutation occurred against the mean probability of the mutation. Dotted line shows relationship based on linear regression. See Supplementary Data 5 for the number of samples with the mutations in question in each cancer type.

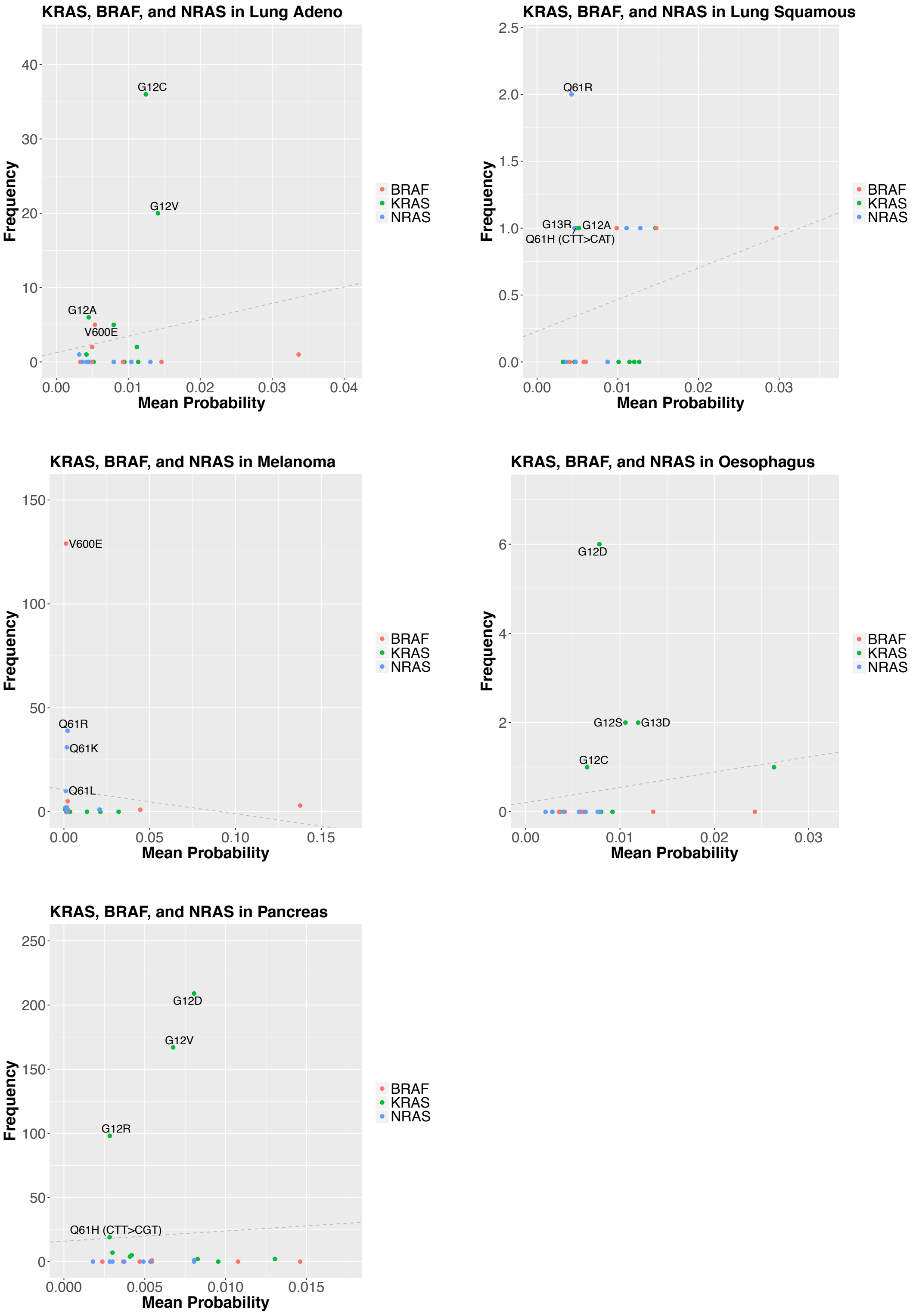

Supplementary Figure 35. Explanation of mutation frequencies by mutation probabilities for *BRAF, KRAS,* and *NRAS* in five additional cancer types

Plots show the number of times each mutation occurred against the mean probability of the mutation. Dotted line shows relationship based on linear regression. See Supplementary Data 5 for the number of samples with the mutations in question in each cancer type.

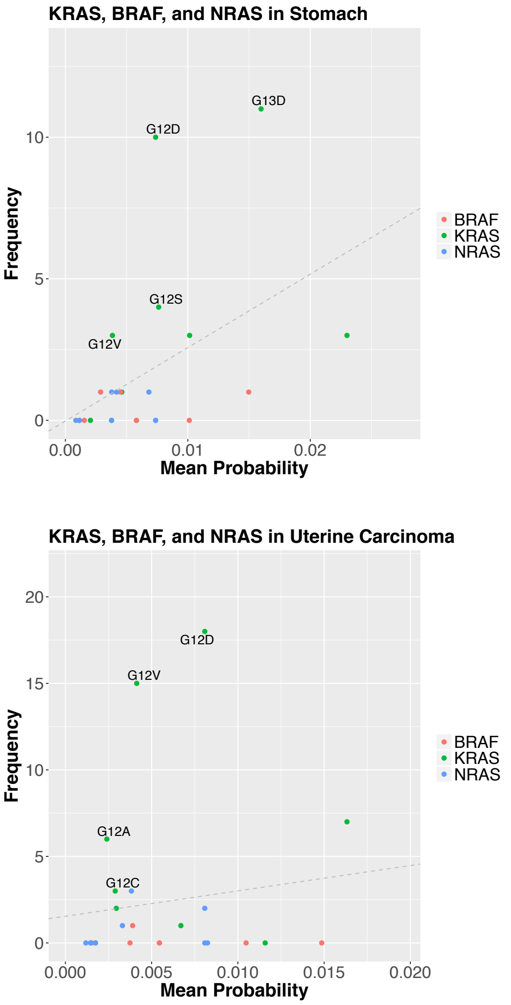

Supplementary Figure 36. Explanation of mutation frequencies by mutation probabilities for *BRAF, KRAS,* and *NRAS* in a final three cancer types

Plots show the number of times each mutation occurred against the mean probability of the mutation. Dotted line shows relationship based on linear regression. See Supplementary Data 5 for the number of samples with the mutations in question in each cancer type.

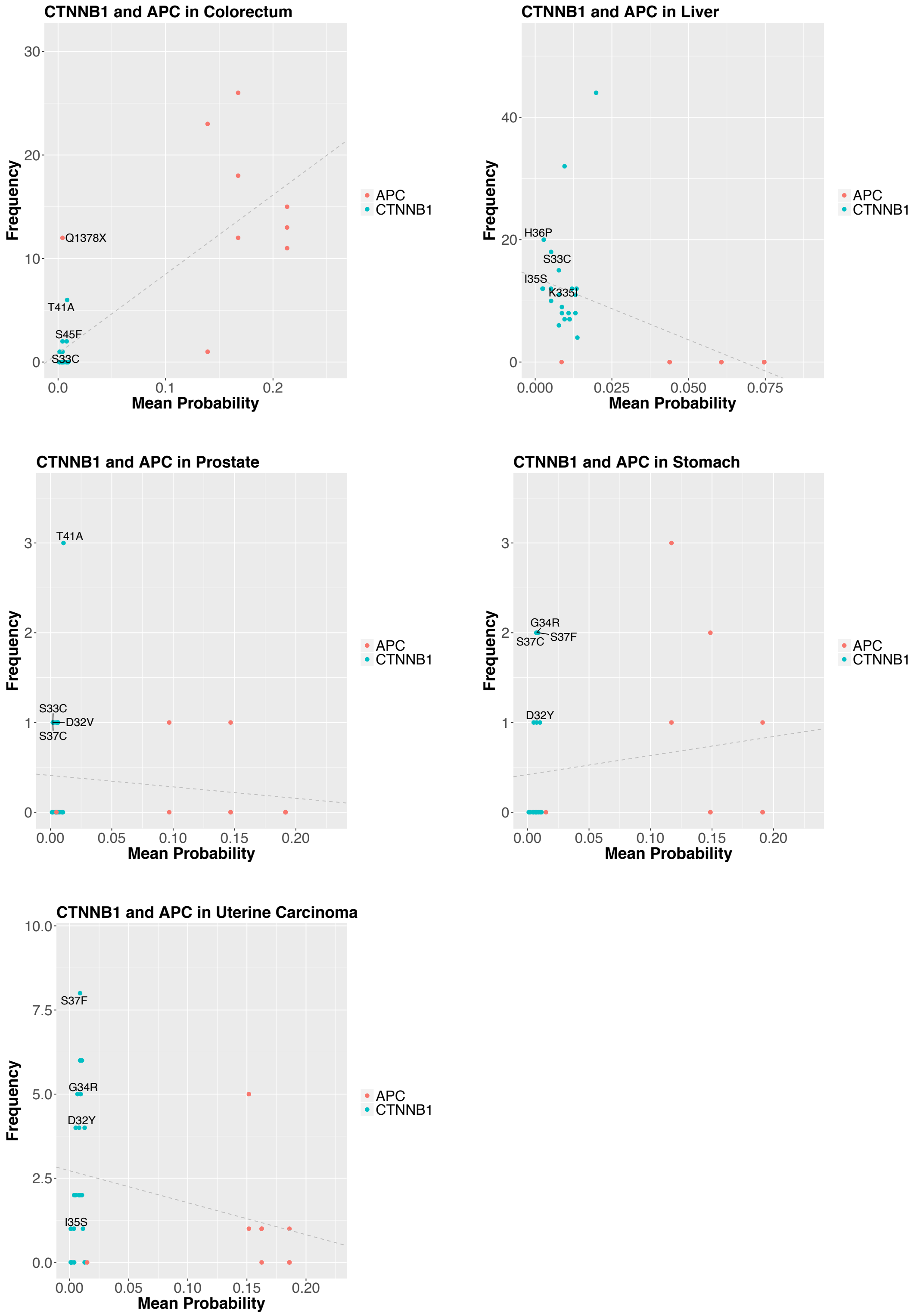

Supplementary Figure 37. Explanation of mutation frequencies by mutation probabilities for *APC* and *CTNNB1* in five cancer types

Plots show the number of times each mutation occurred against the mean probability of the mutation. Dotted line shows relationship based on linear regression. See Supplementary Data 5 for the number of samples with the mutations in question in each cancer type.

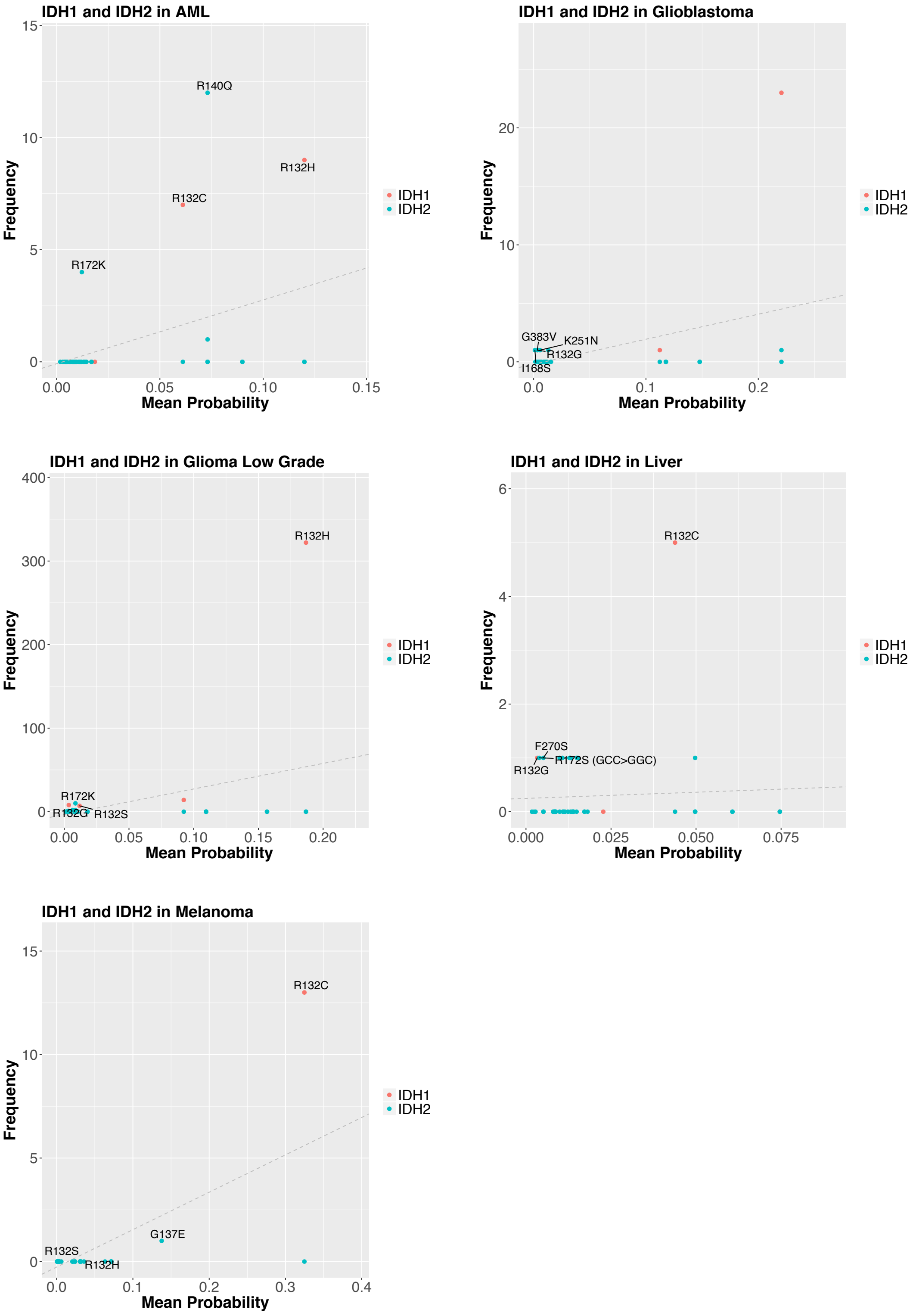

Supplementary Figure 38. Explanation of mutation frequencies by mutation probabilities for *IDH1* and *IDH2* in five cancer types

Plots show the number of times each mutation occurred against the mean probability of the mutation. Dotted line shows relationship based on linear regression. See Supplementary Data 5 for the number of samples with the mutations in question in each cancer type.

Supplementary Tables

Supplementary Table 1. Samples used for study

Disease	Initial Samples	Filtered Samples
AML	394	180
Liver	1,153	1,110
Bladder	515	509
Glioblastoma	396	389
Glioma Low Grade	516	448
Breast	1,107	909
Cervix	198	189
CLL	262	199
Colorectum	531	525
Prostate	741	699
Oesophagus	532	512
Stomach	450	441
Head and Neck	616	610
Thyroid	511	179
Kidney Clear Cell	573	560
Kidney Papillary	282	274
Lung Adeno	230	228
Lung Squamous	497	491
Ovary	380	373
Pancreas	803	718
Melanoma	344	337
Uterine Carcinoma	305	303
