# Supplementary material for: The effects of mutational processes and selection on driver mutations across cancer types
Source: Nat Commun. 2018 May 10;9:1857. doi: 10.1038/s41467-018-04208-6 (PMC5945620; doi:10.1038/s41467-018-04208-6)
Supplement: Supplementary file 3 — Description of Additional Supplementary Files [file 41467_2018_4208_MOESM3_ESM.pdf]

Description of Additional Supplementary Files

File Name: Supplementary Data 1
Description: *Associations between driver mutations and selected mutational signatures within cancer types*

File Name: Supplementary Data 2
Description: *Associations between driver mutations and all mutational signatures within cancer types*

File Name: Supplementary Data 3
Description: *Mutations in each gene considered for differential selection analyses*

File Name: Supplementary Data 4
Description: *Pairs of mutations within a gene with frequencies significantly different from expectation based on mutational signatures*

File Name: Supplementary Data 5
Description: *Pairs of mutations in related genes with frequencies significantly different from expectation based on mutational signatures*

File Name: Supplementary Data 6
Description: *Driver genes used for the study*
